# Vps1 and select autophagic machinery are required for coenzyme Q uptake and trafficking to mitochondria

**DOI:** 10.1016/j.jbc.2026.113592

**Published:** 2026-09-23

**Authors:** Michael D. Guile, Kyle A. Anderson, Akash Jain, Alexandre Toulmay, William A. Prinz, Catherine F. Clarke

**Affiliations:** 1 Department of Chemistry and Biochemistry, University of California, Los Angeles, California, USA; 2 Department of Cell Biology, University of Texas Southwestern Medical Center, Dallas, Texas, USA

**Keywords:** autophagy, dynamin, lipid raft, lipid trafficking, mitochondria, *Saccharomyces cerevisiae*, ubiquinone, vacuole, vps1, yeast

## Abstract

Coenzyme Q (CoQ) is an important lipid found in nearly all cellular membranes in eukaryotes. Biosynthesis of CoQ occurs within mitochondria, where it functions as an electron carrier in oxidative phosphorylation and participates in key metabolic pathways. In both mitochondrial and non-mitochondrial membranes, the hydroquinone form of CoQ (CoQH_2_) also functions as a radical-scavenging antioxidant and participates in other processes required for cell maintenance and survival. Individuals with CoQ deficiency may benefit from high-dose CoQ supplementation; however, its bioavailability is limited, and treatment responses can vary. Here, we sought to gain mechanistic insight into how exogenous CoQ is trafficked to mitochondria. We used the yeast model system *Saccharomyces cerevisiae*, that produce CoQ_6_ with a polyisoprenyl tail containing six isoprene units. A CoQ_6_-deficient (*coq2Δ*) yeast mutant is used to investigate genes and corresponding pathways required for the cellular uptake and trafficking of exogenous CoQ_6_ to mitochondrial respiratory complexes. Specifically, we identify essential residues in the dynamin-like protein Vps1 that are required for CoQ_6_ trafficking and show that yeast *vps1* mutants with known defects in autophagy are incapable of trafficking exogenously supplemented CoQ_6_ to mitochondria. Importantly, we identify a non-canonical role for several autophagic proteins in CoQ_6_ trafficking. Taken together, our data suggest that uptake of exogenous CoQ_6_ and its delivery to the mitochondria relies on a novel, specialized lipid trafficking pathway comprised of select autophagic and endosomal membrane trafficking proteins, and the lytic compartment which serves as a transport hub.

Coenzyme Q (CoQ) is a redox-active lipid with essential roles in mitochondrial metabolism, cellular respiration, and antioxidant protection. It is composed of a fully substituted benzoquinone head group and a polyisoprenyl tail, the length of which is species-dependent (*e*.*g*. CoQ_6_ with six isoprene units in *Saccharomyces cerevisiae* and CoQ_10_ with 10 units in humans). The head group reversibly accepts or donates electrons to interconvert between its reduced form (ubiquinol, CoQH_2_) and oxidized form (ubiquinone, CoQ) (1, 2). At the inner mitochondrial membrane, CoQ plays a vital role in cellular respiration by shuttling electrons from Complex I and Complex II to Complex III (3). These electrons continue down the electron transport chain to produce the proton gradient within mitochondria and ultimately generate ATP *via* oxidative phosphorylation (4). Mitochondrial CoQ is also reduced by several other oxidoreductases, implicating it in key metabolic pathways, such as fatty acid oxidation, branched-chain amino acid catabolism, choline metabolism, and nucleotide biosynthesis (5).

CoQ is synthesized on the matrix side of the inner mitochondrial membrane by a set of biosynthetic proteins and lipid CoQ-intermediates, collectively termed Complex Q in humans or the CoQ synthome in yeast (6, 7, 8). Newly synthesized CoQ is then delivered to respiratory complexes within the mitochondria or exported to non-mitochondrial membranes. While the mitochondrial export pathway of CoQ is still poorly characterized, recent work identified the mitochondrial proteins Cqd1 and Cqd2 as novel regulators of mitochondrial CoQ distribution (9, 10). Additionally, growing evidence suggests that membrane contact sites between mitochondria and the endoplasmic reticulum may play a critical role in CoQ transport (11, 12, 13).

In non-mitochondrial membranes, CoQ plays pivotal roles in redox regulation and antioxidant protection. For example, CoQH_2_ mitigates lipid peroxidation of cellular membranes by quenching of lipid peroxyl radicals (14). Moreover, CoQ interacts with other antioxidants; CoQ can be reduced by ascorbate to CoQH_2_, and CoQH_2_ can in turn reduce the alpha-tocopheroxyl radical to regenerate alpha-tocopherol (15). CoQ also participates in the plasma membrane redox system to combat oxidative stress and maintain cell bioenergetics (16, 17), with a growing number of studies highlighting the importance of CoQH_2_ at the plasma membrane in suppression of ferroptosis (18, 19, 20).

Defects in CoQ_10_ biosynthesis in individuals result in a spectrum of disease phenotypes (21, 22). Primary CoQ_10_ deficiencies are a group of genetic diseases caused by mutations in genes encoding CoQ_10_ biosynthesis proteins, while secondary CoQ_10_ deficiency is more common and is caused by other factors that disrupt CoQ_10_ biosynthesis, such as statin treatment, ageing, and mitochondrial disease (23, 24). CoQ_10_ deficiency can result in encephalopathy, cardiomyopathy, liver failure, muscular myopathy, and other clinical manifestations. Individuals may take high-dose oral CoQ_10_ supplements to ameliorate symptoms, but there are many challenges with this approach, such as low bioavailability, high variability in response among individuals, and inconsistent uptake based on supplement formulation (25, 26, 27, 28).

*Saccharomyces cerevisiae*, or baker’s yeast, is an important model system for studying the biosynthesis and uptake of CoQ. Knockout of the early biosynthetic gene *COQ2*, a polyisoprenyl transferase, completely abolishes CoQ_6_ biosynthesis and prevents the accumulation of toxic CoQ_6_ intermediates (29). As such, the *COQ2* deletion mutant (*coq2Δ*) mutant lacks endogenous CoQ_6_, is unable to respire, and thus fails to grow in medium containing a non-fermentable carbon source, such as glycerol (YPG). Supplementation with exogenous CoQ_6_ restores respiration and partially rescues growth of *coq2Δ* in YPG. Previous work examined a series of mutants that harbored gene deletions (*orfΔ*) in conjunction with *coq2Δ* to identify essential genes for the uptake and trafficking of exogenous CoQ_6_ (28, 29, 30).

The *orfΔcoq2Δ* model system was used to interrogate candidate genes required for efficient uptake and trafficking of exogenous CoQ_6_, positing that CoQ_6_ trafficking occurs *via* the endomembrane system (29, 31, 32). Notably, *VPS1* was identified as an essential gene for the rescue of the *coq2Δ* mutant mediated by exogenous CoQ_6_ (29). Vps1 is a dynamin-like protein involved in membrane fusion and fission events at multiple cellular membranes, such as endocytosis, endomembrane trafficking, and peroxisome fission (33). More recent work identified a role for Vps1 in autophagy (34, 35).

Macroautophagy (hereafter referred to as autophagy) is an intracellular process in which components of the cytosol are sequestered within double-membrane vesicles termed autophagosomes and delivered to the lytic compartment (lysosome in mammalian cells, vacuole in yeast) for degradation and subsequent recycling of macromolecular constituents (36, 37). Autophagy maintains cell energetics during periods of starvation and in response to various stress conditions. The core machinery is highly conserved from yeast to humans. Over 30 autophagic proteins have been identified in yeast, and the core machinery are categorized into four functional groups— {1} The Atg1 complex (ULK1/2 complex in mammals), {2} Atg9 and its cycling system, {3} the PtdIns 3-kinase (PI3K) complex, and {4} two ubiquitin-like (Ubl) conjugation systems (38, 39, 40). Some core autophagic machinery has been found to have non-canonical roles in other membrane trafficking events, such as specialized endocytic and secretory pathways (41, 42, 43, 44).

Given that the processes of CoQ cellular uptake and trafficking remain poorly understood (28), studies aimed at elucidating the mechanisms of CoQ uptake and intracellular distribution are vital to better understand the essential role of CoQ in antioxidant protection, metabolism, and cellular energetics. In this study, we characterized the role of Vps1 in mediating the rescue by exogenous CoQ_6_ and identified several autophagic proteins with essential roles in CoQ_6_ trafficking. We showed that *vps1* point mutants with defects in autophagy are also defective in CoQ_6_ trafficking. Additionally, we identified *ATG6*, *ATG8*, *ATG9*, *ATG11*, *ATG12*, *ATG16*, *PEP4*, *VPS4*, and *VPS38* as essential genes for efficient CoQ_6_ trafficking. Strikingly, other core autophagy proteins are not required, indicating that canonical autophagy is dispensable for CoQ_6_ uptake and trafficking to mitochondria. These data suggest that a specialized pathway exists for uptake and trafficking of CoQ that requires only a subset of known autophagic proteins. To our knowledge, this is the first study to identify a role for autophagic machinery in the cellular uptake and distribution of CoQ.

## Results

### Vps1 is required for efficient trafficking of exogenous CoQ_6_ to mitochondria

We first confirmed the finding that identified *VPS1* as an essential gene for trafficking of exogenous CoQ_6_ to mitochondrial respiratory complexes (29). Growth of the *vps1Δ* single mutant and *vps1Δcoq2Δ* double mutant was monitored over seven days in YPG supplemented with 2 μM CoQ_6_ or ethanol as a vehicle control. As described previously, supplementation of YPG medium with exogenous CoQ_6_ partially restores growth of the *coq2Δ* mutant (Fig. 1, *A* and *B*). In contrast, growth of the *vps1Δcoq2Δ* double mutant in YPG could not be rescued by the addition of exogenous CoQ_6_, confirming that deletion of *VPS1* disrupts delivery of exogenous CoQ_6_ to mitochondrial respiratory complexes. Although the *vps1Δcoq2Δ* mutants failed to grow on YPG supplemented with CoQ_6_, the yeast cells retained viability as demonstrated by the serial plate dilution (Fig. 1*C*). We next quantified whole-cell CoQ_6_ content by LC-MS/MS of cells grown in YPG with or without exogenous CoQ_6_ after seven days. Surprisingly, the *vps1Δcoq2Δ* mutant had a 20-fold increase in CoQ_6_ content compared to the *coq2Δ* mutant (Fig. 1*D*), despite the failure to grow (Fig. 1, *A* and *B*). Quantification at earlier time points through the growth period show an elevated CoQ_6_ content in the *vps1Δcoq2Δ* mutant at three and five days (Fig. 1*E*). It is notable that the *vps1Δ* single mutant, which retains the ability to synthesize CoQ_6_, nevertheless accumulates a higher CoQ_6_ content than does WT when cultured in the presence of exogenous CoQ_6_ (Fig. 1*D*). This observation indicates that the defect in the trafficking pathway that handles exogenous CoQ_6_ in the *vps1Δ* mutant persists despite the presence of endogenous CoQ_6_ biosynthesis.

**Figure 1:**
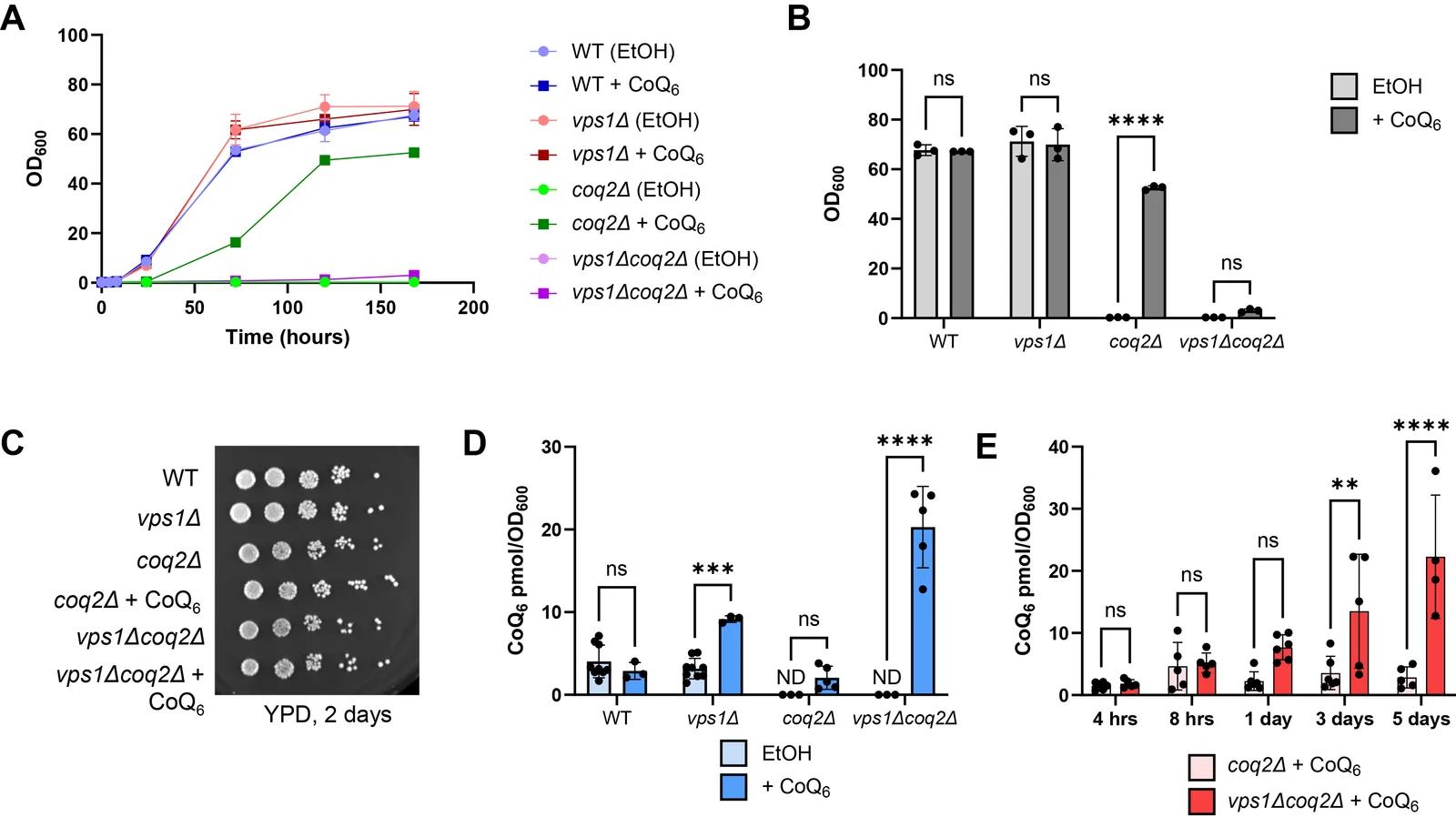
**Vps1 is required for CoQ_6_ trafficking to mitochondrial respiratory complexes.***A*) The OD_600_ of strains grown in medium requiring respiration (YPG) with or without 2 μM CoQ_6_ was measured over seven days. *B*, the terminal OD_600_ after seven days of growth from *panel A* is shown. *C*, seven days of growth as described in *panel A*, cells were serially diluted and spot-plated onto fermentable medium (YPD) and then incubated for two days to verify viability. One of three biological replicates is shown. *D*, the cell-associated CoQ_6_ content was determined from lipid extracts of whole cells after seven days of growth as described in *panel A via* HPLC-MS. *E*, the cell-associated CoQ_6_ content was determined at the indicated times after growth as described in *panel A*. Statistics were determined *via* two-way ANOVA with Šidák’s multiple comparisons test. ∗, *p* ≤ 0.05; ∗∗, *p* ≤ 0.01; ∗∗∗, *p* ≤ 0.001; and ∗∗∗∗, *p* ≤ 0.0001; ns, *p* > 0.05; ND indicates CoQ_6_ content was below detection limit.

To determine if the accumulation of exogenous CoQ_6_ in the *vps1Δcoq2Δ* mutant is dependent on the metabolic state, cells were grown in rich medium containing dextrose as a fermentable carbon source (YPD), with or without exogenous CoQ_6_ for seven days. Supplementation with exogenous CoQ_6_ in YPD improved growth of the *coq2Δ* single mutant to a greater degree than that of the *vps1Δcoq2Δ* double mutant (Fig. S1, *A* and *B*). All strains retained viability (Fig. S1*C*). Exogenous CoQ_6_ was detected at four hours and accumulated to significantly higher levels in the *vps1Δcoq2Δ* mutant than in the *coq2Δ* mutant (Fig. S1, *D* and *E*). This suggests that the accumulation of CoQ_6_ observed for the *vps1Δcoq2Δ* mutant is independent of metabolic state. Curiously, in yeast cultured in YPD, the endogenous CoQ_6_ content is decreased in the *vps1Δ* single mutant when compared to WT (in the absence of exogenous CoQ_6_). Taken together, these results demonstrate that the deletion of *VPS1* creates a trafficking defect in which exogenous CoQ_6_ accumulates but is inaccessible to the mitochondrial respiratory complexes, highlighting a critical role for Vps1 in the distribution of exogenously supplied CoQ_6_.

### CoQ_6_ transport to and from the plasma membrane is impaired in the *vps1Δ* mutant

In order to determine the cellular location where CoQ_6_ may be accumulating, we quantified the content of CoQ_6_ in highly enriched subcellular fractions containing yeast plasma membrane. Cell homogenate underwent renografin density gradient centrifugation with fractions 9 to 11 corresponding to plasma-membrane enriched membranes and fractions 4 to 6 containing other membranes, including ER, mitochondrial, and vacuolar membranes (Fig. 2*A*). The subcellular fractions were isolated from cells cultured overnight in YPD. As shown in Figure 2*B*, the *vps1Δ* single mutant has only 60% as much endogenous CoQ_6_ in the plasma membrane fraction as compared to WT. This result indicates that the deletion of *VPS1* impairs the distribution of endogenous CoQ_6_ originating from its site of synthesis in the mitochondria to the plasma membrane. We also analyzed the content of CoQ_6_ present in the plasma membrane fractions isolated from either the *coq2Δ* single mutant or the *vps1Δcoq2Δ* double mutant following YPD culture in the presence of exogenous CoQ_6_ (Fig. 2*C*). Intriguingly, the *vps1Δcoq2Δ* double mutant contained 40% more exogenous CoQ_6_ in the plasma membrane fraction when compared to the same plasma membrane fraction isolated from the *coq2Δ* single mutant. These results suggest that in the absence of Vps1, a portion of exogenous CoQ_6_ that is taken up accumulates in the plasma membrane. Moreover, exogenous CoQ_6_ content in fractions 4 to 6 was 50% higher in the *vps1Δcoq2Δ* double mutant than that of the *coq2Δ* single mutant, suggesting exogenous CoQ_6_ accumulates in additional membranes. Taken together, these alterations in plasma membrane content of CoQ_6_ suggest that Vps1 plays a crucial role in the trafficking of exogenous CoQ_6_ and is also an important player in the distribution of endogenously synthesized CoQ_6_ in the mitochondria to the plasma membrane.

**Figure 2:**
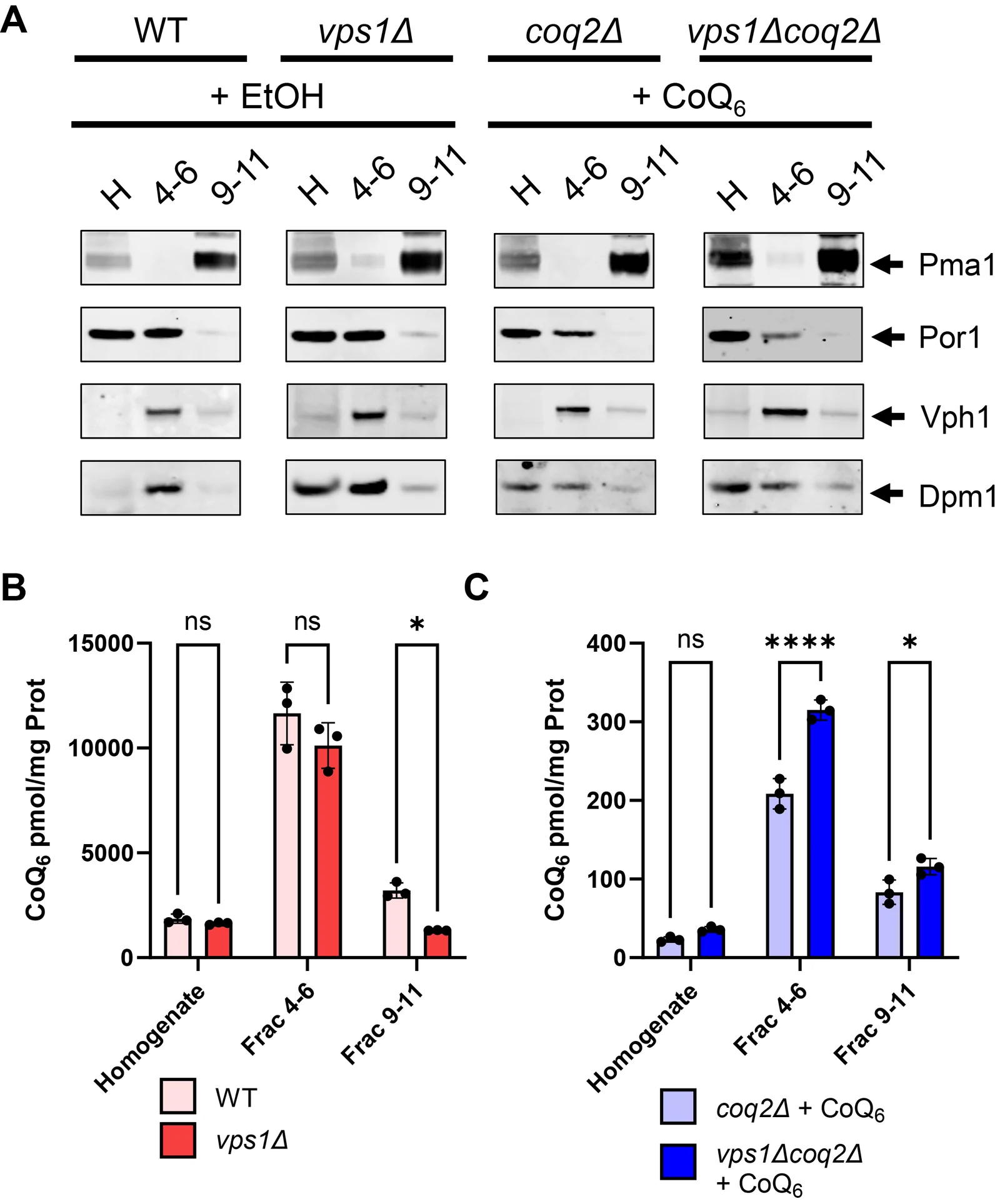
**Vps1 participates in subcellular distribution of CoQ_6_.***A*, subcellular fractions were prepared using density gradient centrifugation from a whole-cell homogenate (H) after overnight growth in YPD with 2 μM CoQ_6_ or ethanol (EtOH) as a vehicle control. Fractions 9 to 11 are enriched in plasma membrane, while fractions 4 to 6 contained most of other cellular membranes. The plasma membrane extraction quality was checked by immunoblotting. A representative immunoblot (one of three biological replicates) is shown. Antibodies to marker proteins included, Pma1, plasma membrane P2-type H+-ATPase; Por1, mitochondrial porin; Vph1, subunit A of the vacuolar-ATPase V0 domain; Dpm1, dolichol phosphate mannose synthase of the ER membrane. *B*, the CoQ_6_ content was determined from lipid extracts of cell homogenate or indicated fractions from cultures grown in YPD with ethanol as the vehicle control. CoQ_6_ content was determined using HPLC-MS. *C*, the CoQ_6_ content was determined from lipid extracts as described in *panel B* from cultures grown in YPD containing CoQ_6_. Statistics were determined *via* two-way ANOVA with Šidák’s multiple comparisons tests. ∗, *p* ≤ 0.05; ∗∗, *p* ≤ 0.01; ∗∗∗, *p* ≤ 0.001; and ∗∗∗∗, *p* ≤ 0.0001; ns, *p* > 0.05; ND indicates CoQ_6_ content was below detection limit.

### Vps1 residues essential for autophagy are also essential for efficient CoQ_6_ trafficking

Vps1 is involved in many membrane fusion and fission processes throughout the cell and has a recently discovered role in autophagy (33, 34, 35). To determine which roles of Vps1 are essential for trafficking of exogenous CoQ_6_, we utilized a set of previously characterized *vps1* point mutants that span the different domains of Vps1 (Fig. 3*A*) and thus affect specific functions of Vps1 ((34, 35, 45, 46, 47, 48, 49, 50) Table 1). The *vps1Δcoq2Δ* double mutant was transformed with plasmids expressing each individual *vps1* point mutant, wild-type (WT) *VPS1*, or an empty vector (EV) control. Immunoblot analyses of whole-cell lysates showed that all *vps1Δcoq2Δ* transformants expressing the *vps1* point mutants have normal Vps1 polypeptide content when compared with the WT *VPS1* control (Fig. 3, *B* and *C*). We next assessed growth in YPG medium with or without exogenous CoQ_6_ (Figs. 3*D* and S2). Expression of several *vps1* point mutants (*E461K*, *P564A*, *S599V*, and *KKK591-593AAA*) partially rescued growth of *vps1Δcoq2Δ*, indicating that these point mutations do not impact CoQ_6_ trafficking (Fig. 3*D*). In contrast, expression of either *vps1*-*T63A* or *vps1-I649K* failed to restore growth of *vps1Δcoq2Δ*. Quantification of CoQ_6_ content after seven days of growth showed that only the *vps1* point mutants that failed to restore growth of *vps1Δcoq2Δ* (*vps1-T63A*, *vps1-I649K*), and the empty-vector control accumulated increased amounts of exogenous CoQ_6_ (Fig. 3*E*). These results indicate that yeast expressing *vps1-T63A* or *vps1-I649K* showed impaired trafficking of exogenous CoQ_6_ to the mitochondrial respiratory complexes.

**Figure 3:**
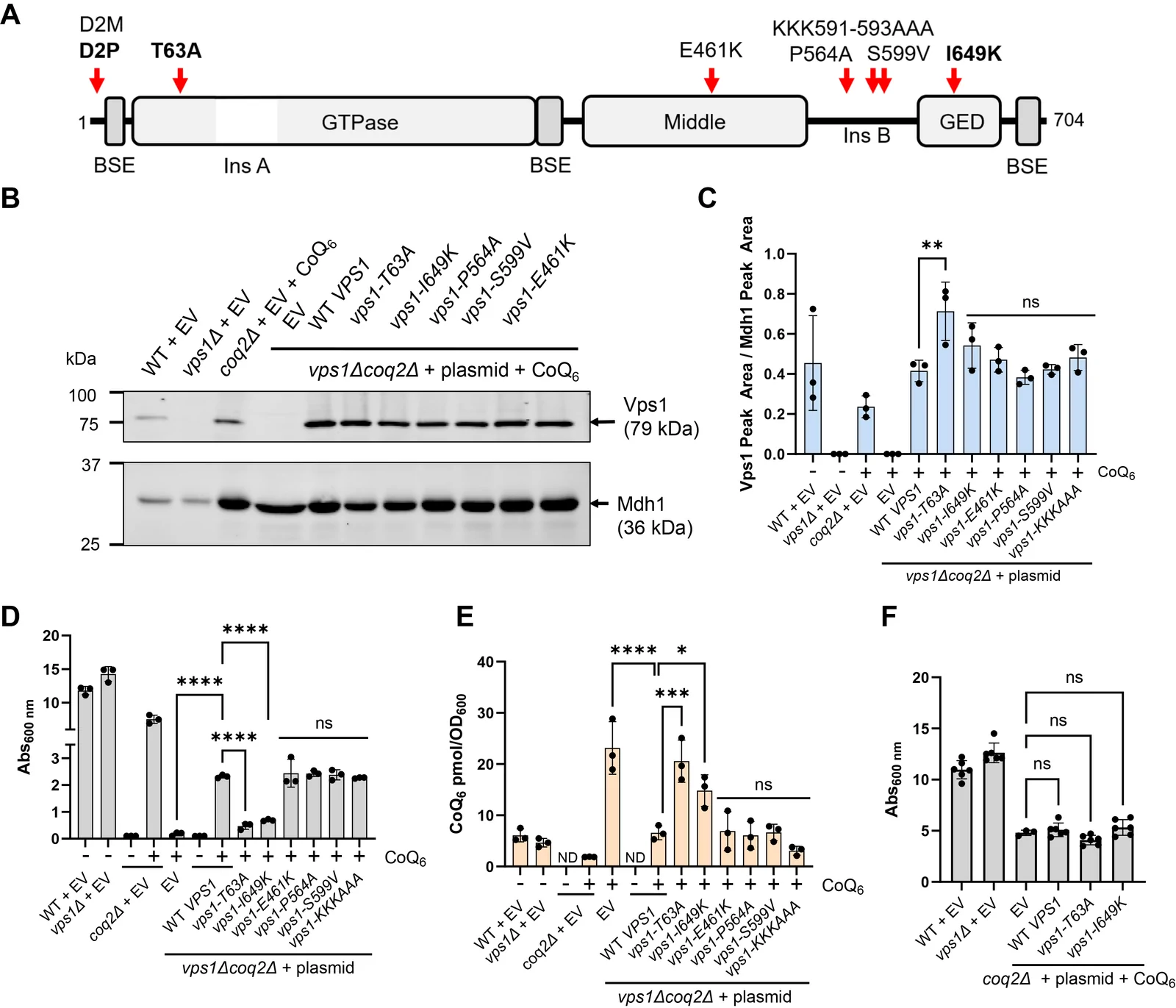
**Yeast expressing *vps1-T63A* or *vps1-I649K* show impaired trafficking of exogenous CoQ_6_ to respiratory complexes.***A*, diagram showing the domain organization of *S. cerevisiae* Vps1, including bundle signaling elements (BSE), GTPase domain, Insert A (Ins A), the Middle domain, Insert B (Ins B), and the GTPase effector domain (GED). *Red arrows* denote mutants investigated in this study. Bold text indicates mutants that fail to efficiently deliver exogenous CoQ_6_ to respiratory complexes. *B*, aliquots of whole-cell lysates containing 25 μg protein were subjected to 12% Tris-glycine SDS-PAGE, transferred to a PVDF membrane, and probed with antisera to Vps1 or malate dehydrogenase (Mdh1) (to provide a loading control). A representative immunoblot (one of three biological replicates) is shown. *C*, band intensities corresponding to Vps1 were quantified by hand using ImageJ, were normalized to Mdh1, and plotted as a ratio of Vps1 peak area/Mdh1 peak area. The data show the mean ± the standard deviation of three biological replicates. *D*, the Abs_600 nm_ of strains grown in YPG + 0.5% dextrose (YPGG) with or without 2 μM CoQ_6_ was measured after seven days. *E*, the cell-associated CoQ_6_ content was determined from lipid extracts of whole cells after seven days of growth in YPGG with or without 2 μM CoQ_6_. CoQ_6_ content was determined using HPLC-MS. *F*, the Abs_600 nm_ of the *coq2Δ* single deletion strain harboring the designated plasmids were grown in YPGG with or without 2 μM CoQ_6_ was measured after seven days under identical conditions as *panel D*. Statistics were determined *via* one-way ANOVA with Dunnett’s multiple comparisons test. ∗, *p* ≤ 0.05; ∗∗, *p* ≤ 0.01; ∗∗∗, *p* ≤ 0.001; and ∗∗∗∗, *p* ≤ 0.0001; ns, *p* > 0.05; ND indicates levels were below detection limit.

**Table 1:** *vps1* point mutants investigated in this study

Point mutation	Description
D2M	Mutation at the N-terminus that allows the residue to be acetylated by multiple subclasses of acetyltransferases; autophagy functions normally (35)
D2P	Mutation at the N-terminus that abolishes acetylation; disrupted autophagy as seen by reduced interaction with Vam3, which prevents fusion of the autophagosome with the vacuole (35)
T63A	Mutation in GTPase domain; impaired endomembrane trafficking such as fragmented vacuolar morphology and fewer endosomes and decreased endosomal recycling to the plasma membrane; defective in peroxisome fission in the *dnm1Δ* background; decreased autophagy as seen by decreased Pho8Δ60 activity and increased puncta of mislocalized Atg9-GFP; corresponds to DYN1p.Thr65Ala in human Dnm1 (34, 45)
E461K	Mutation in proposed actin-binding α helix of the central stalk; mutant oligomerizes and binds actin but fails to form double-ringed structures; disrupted early endocytosis such as decreased recruitment of endocytic proteins and fewer invagination sites resulting in delayed uptake of lucifer yellow dye (34, 46, 47)
P564A	Mutation in the SH3 binding motif in the insert B region of the GTPase effector or self-assembly domain (GED); mutant is unable to self-oligomerize; disrupted endocytosis such as reduced interaction with Rvs167, and extended endocytic invagination and reduced scission at endocytic sites (48)
S599V	Mutation of a serine in the GED to abolish phosphorylation; disrupted endocytosis such as decreased endocytosis of Snc1 at the plasma membrane, inhibited interaction with Rvs167, and extended endocytic invagination and reduced scission at endocytic sites (34, 49)
KKK591–593AAA (KKKAAA)	Mutation of conserved lysine residues in the insert B region of the GED; disrupted lipid-binding to PI(4,5)P2; disrupted endocytosis such as decreased scission and increased retraction of endocytic invaginations at the plasma membrane (50)
I649K	Mutation in the GED; disrupted endocytosis such as decreased endocytosis of Snc1 at the plasma membrane; unable to self-assemble to tubulate liposomes; disrupted autophagy as seen by decreased Pho8Δ60 activity and increased puncta of mislocalized Atg9-GFP; corresponds to DYN1 p.Ile690Lys mutation in human Dnm1 (34, 45)

The yeast *vps1-T63A* and *vps1-I649K* mutants are homologous to the human DYN1 p.Thr65Ala (51) and the DYN1 p.Ile690Lys mutants (52), the latter of which have been classified as dominant-negative mutations within the context of endocytosis. To determine whether *vps1-T63A* and *vps1-I649K* exert dominant-negative effects on CoQ_6_ trafficking, we co-expressed each *vps1* point mutant in the *coq2Δ* background, which has the genomic *VPS1* allele. Expression of either *vps1-T63A* or *vps1-I649K* did not significantly alter the growth rescue by exogenous CoQ_6_ in YPG medium (Fig. 3*F*). This suggests that *vps1-T63A* and *vps1-I649K* do not act as dominant negative alleles with respect to CoQ_6_ trafficking.

Recent work has identified an essential role for Vps1 in autophagosome formation and fusion to the vacuole (34, 35). To perform this function, the N-terminus of Vps1 must undergo acetylation by Nat3, a member of the NatB class of N-terminal acetyltransferase (53) and an essential protein for CoQ_6_ uptake and transport (29). Deletion of *NAT3* or mutation of the second Vps1 residue to a proline (Vps1-D2P) results in a complete block in autophagy, while mutation of this residue to a methionine (Vps1-D2M) allows for constitutive acetylation by other classes of N-terminal acetyltransferases with no impact on autophagy (Table 1) (35).

To determine whether defects in autophagy may interfere with trafficking of exogenous CoQ_6_, we expressed either *vps1-D2M* or *vps1-D2P* in the *vps1Δcoq2Δ* double mutant and monitored growth in YPG medium with or without exogenous CoQ_6_ over seven days. Immunoblot analyses of whole-cell lysates showed that *vps1Δcoq2Δ* expressing *vps1-D2M or vps1-D2P* had Vps1 polypeptide content comparable to the WT *VPS1* control (Fig. 4, *A* and *B*). Expression of WT *VPS1* or *vps1-D2M* partially restored growth of the *vps1Δcoq2Δ* mutant in YPG with exogenous CoQ_6_ (Figs. 4*C* and S2). In contrast, expression of *vps1-D2P* failed to rescue growth, indicating that N-terminal acetylation of Vps1 is required for efficient CoQ_6_ trafficking. Notably, expression of either *vps1-D2M* or *vps1-D2P* produced whole-cell CoQ_6_ levels similar to that of the WT *VPS1* control (Fig. 4*D*). Neither mutant exhibited dominant-negative effects on CoQ_6_ uptake and trafficking (Fig. 4*E*). To summarize, we have identified three *vps1* point mutants that are unable to restore efficient CoQ_6_ trafficking in the *vps1Δcoq2Δ* mutant: *vps1-T63A*, *vps1-I649K*, and *vps1-D2P*. Given that all three of these Vps1 point mutations impact the autophagic function of Vps1, this suggests a functional overlap between autophagy and the trafficking of exogenous CoQ_6_.

**Figure 4:**
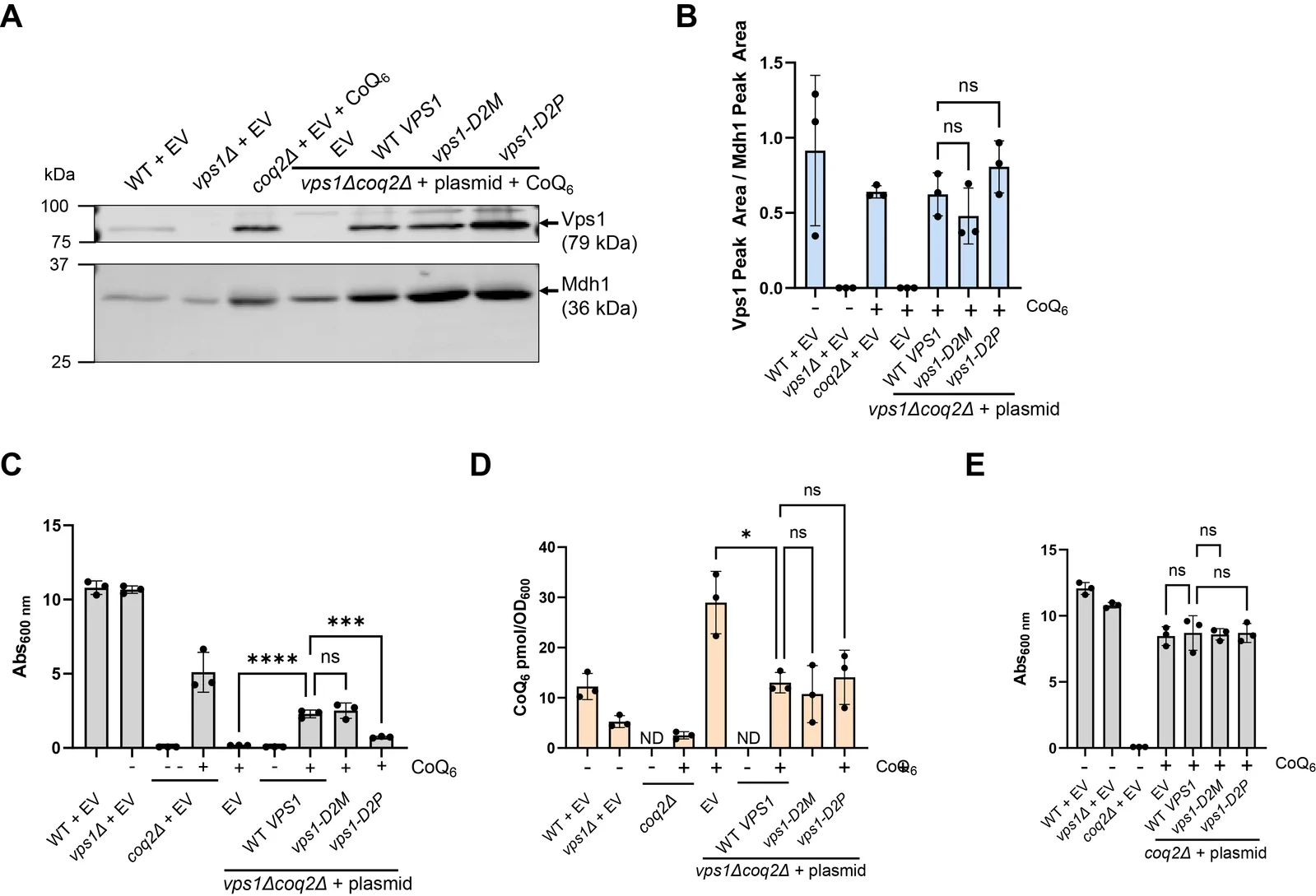
**The *vps1-D2P* mutant impairs trafficking of exogenous CoQ_6_ to respiratory complexes.***A*, Aliquots of whole-cell lysates containing 25 μg protein were subjected to 12% Tris-glycine SDS-PAGE, transferred to a PVDF membrane, and probed with antisera to Vps1 or Mdh1 (to provide a loading control). A representative immunoblot (one of three biological replicates) is shown. *B*, band intensities corresponding to Vps1 were quantified by hand using ImageJ, were normalized to Mdh1, and plotted as a ratio of Vps1 peak area/Mdh1 peak area. The data show the mean ± the standard deviation of three biological replicates. *C*, the Abs_600 nm_ of strains grown in YPGG with or without 2 μM CoQ_6_ was measured after seven days. *D*, the cell-associated CoQ_6_ content was determined from lipid extracts of whole cells after seven days of growth in YPGG with or without 2 μM CoQ_6_. CoQ_6_ content was determined using HPLC-MS. *E*) The Abs_600 nm_ of the *coq2Δ* single deletion strain harboring the designated plasmids were grown in YPGG with or without 2 μM CoQ_6_ was measured after seven days under identical conditions as *panel C*. Statistics were determined *via* one-way ANOVA with Dunnett’s multiple comparisons test. ∗, *p* ≤ 0.05; ∗∗, *p* ≤ 0.01; ∗∗∗, *p* ≤ 0.001; and ∗∗∗∗, *p* ≤ 0.0001; ns, *p* > 0.05; ND indicates levels were below detection limit.

To verify the autophagy phenotype of our *vps1Δ* and *vps1Δcoq2Δ* mutants, cells were transformed with a plasmid expressing a GFP-Atg8 recombinant protein. When autophagy is induced by culturing in a nitrogen-starvation medium (SD–N), the recombinant protein is delivered *via* autophagosomes to the lytic compartment for degradation. Cleaved GFP is resistant to lytic degradation, enabling its detection *via* immunoblotting (54, 55). Immunoblots showed the presence of cleaved GFP in WT, *vps1Δ*, and *vps1Δcoq2Δ*, but not the *atg1Δ* autophagy-deficient control (Fig. S3*A*). Densitometric analyses showed that the ratio of cleaved GFP to total GFP was significantly reduced in *vps1Δ* and *vps1Δcoq2Δ* mutants when compared with WT, confirming previous observations that autophagy is significantly impaired in *vps1* mutants (Fig. S3*B*) (34, 35).

### Nat3 acetylates multiple proteins in the CoQ_6_ uptake and trafficking pathway

Previous work showed that expression of *vps1-D2M* in the *nat3Δ* strain was unable to restore autophagy, suggesting that other autophagic proteins are acetylated by Nat3 (35). To investigate whether the role of Nat3 acetylation in CoQ trafficking extends beyond the acetylation of Vps1, we transformed *nat3Δcoq2Δ* with plasmids expressing either the *vps1-D2M* or the *vps1-D2P* point mutant and appropriate controls. Expression of WT *NAT3* partially rescued growth, which is likely due to differences in gene dosage and plasmid stability (56). Expression of either *vps1-D2M* or *vps1-D2P* in the *nat3Δcoq2Δ* double mutant failed to restore growth in YPG supplemented with CoQ_6_ (Fig. 5*A*), indicating that N-terminal acetylation of other proteins by Nat3 is required for CoQ_6_ trafficking. Consistent with a requirement for Nat3 acetylation for CoQ_6_ trafficking, the whole-cell CoQ_6_ content was significantly higher in the *nat3Δcoq2Δ* double mutant when compared with *coq2Δ* (Fig. 5*B*), recapitulating the phenomenon of high CoQ_6_ content observed in the *vps1Δcoq2Δ* double mutant.

**Figure 5:**
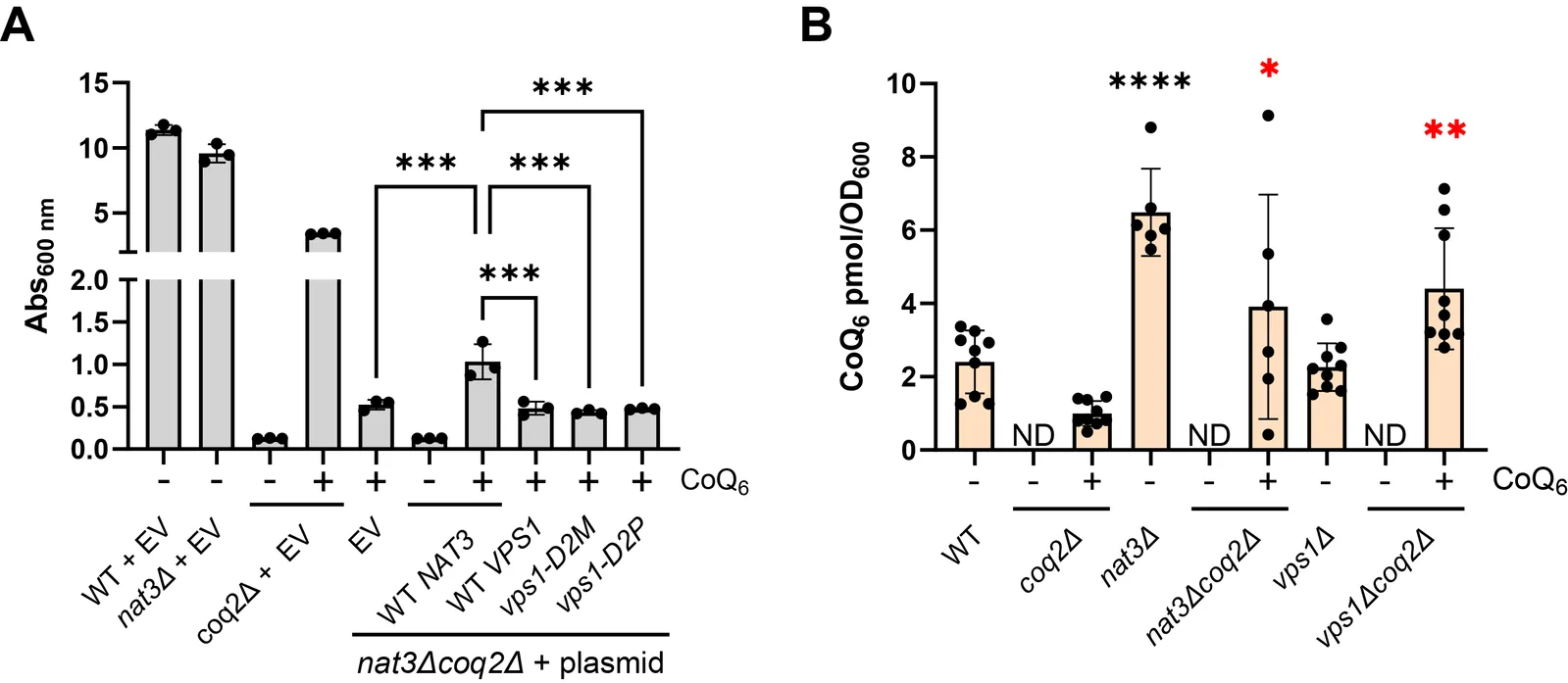
**Nat3 acetylation of other proteins besides Vps1 is required for efficient CoQ_6_ trafficking.***A*, the Abs_600 nm_ of strains grown in YPGG with or without 2 μM CoQ_6_ was measured after seven days. *B*, the cell-associated CoQ_6_ content was determined from lipid extracts of whole cells after three days of growth in YPD with or without 2 μM CoQ_6_. CoQ_6_ content was determined using HPLC-MS. Statistics were determined *via* one-way ANOVA with Dunnett’s multiple comparisons test. ∗, *p* ≤ 0.05; ∗∗, *p* ≤ 0.01; ∗∗∗, *p* ≤ 0.001; and ∗∗∗∗, *p* ≤ 0.0001; ns, *p* > 0.05; ND indicates levels were below detection limit. *Black asterisks* denote significance of comparison to WT and red asterisks denote significance of comparison to *coq2Δ* with exogenous CoQ_6_.

### Select autophagic machinery is essential for uptake and transport of CoQ

Six genes were previously identified as essential for the rescue of the *coq2Δ* mutant mediated by exogenously provided CoQ_6_ (*CDC10*, *NAT3*, *RTS1*, *RVS161*, *RVS167*, and *VPS1*) (29). Several of these genes have functional roles within autophagy (45, 57, 58, 59, 60, 61). To further investigate the connection between autophagy and CoQ_6_ uptake and trafficking, we screened a set of autophagic genes using the *orfΔcoq2Δ* model system (Table 2). Genes were selected based on the criteria that the corresponding single deletion mutant had a complete block in autophagy and disrupted either different components of the core autophagic machinery or a type of selective autophagy, such as pexophagy, mitophagy, or lytic degradation within the vacuole. To verify that the lack of respiratory growth observed for each autophagic *orfΔcoq2Δ* mutant was solely due to lack of CoQ_6_, all *orfΔcoq2Δ* mutants were functionally complemented with *COQ2* and screened for restored growth on YPG medium with 0.05% dextrose (YPGG) (Fig. S4). Additionally, we assessed the capacity for autophagy in each of the selected *orfΔcoq2Δ* mutants using the GFP-Atg8 cleavage assay (Fig. S5).

**Table 2:** Yeast autophagic gene candidates selected for screening

Yeast gene	Human homolog	Role in autophagy
*ATG1*	ULK1	Protein serine/threonine kinase; required for vesicle formation in autophagy (38)
*ATG2*	ATG2A, ATG2B	Putative lipid transport protein; peripheral membrane protein required for vesicle formation during autophagy (38)
*ATG4*	ATG4A, ATG4B, ATG4C, ATG4D	Conserved cysteine protease required for autophagy; cleaves Atg8 to a form required for autophagosome generation (38)
*ATG6/VPS30*	BECN1	Subunit of PtdIns 3-kinase complexes I and II; Complex I is essential in autophagy, Complex II is required for vacuolar protein sorting (38, 107)
*ATG7*	ATG7	Dual specificity member of the E1 family; mediates the attachment of Atg12p to Atg5p and Atg8p to phosphatidylethanolamine which are required steps in autophagosome formation (38)
*ATG8*	LC3	Ubiquitin-like protein conjugated to phosphatidylethanolamine; role in membrane fusion and phagophore expansion during autophagosome formation; deconjugation is also required for autophagosome biogenesis (38)
*ATG9*	ATG9A, ATG9B	Lipid scramblase that mediates autophagosomal membrane expansion (38, 39)
*ATG11*	ATG11	Scaffold protein involved in localizing proteins to the PAS; role in mitophagy, nucleophagy, reticulophagy, pexophagy and the cytoplasm to vacuole (CVT) targeting pathway (62)
*ATG12*	ATG12	Ubiquitin-like modifier involved in autophagy; conjugated to Atg5 to form a complex involved in Atg8 lipidation (38)
*ATG16*	ATG16L1	Interacts with Atg12-Atg5 conjugates to form Atg12-Atg5-Atg16 multimers, which bind to membranes and localize to the pre-autophagosomal structure and are required for autophagy (38)
*ATG32*	BCL2L13	Mitochondrial outer membrane protein required to initiate mitophagy; recruits the Atg11 and Atg8 to the mitochondrial surface to initiate mitophagy (38)
*ATG36*	NRDE	Pex3 interacting protein, required for pexophagy; interacts with Atg8 and Atg11 (38)
*PEP4*	CTSD	Vacuolar aspartyl protease (proteinase A); required for post-translational precursor maturation of vacuolar proteinases (108)

Descriptions are from the *Saccharomyces* genome database https://www.yeastgenome.org.

Seven autophagic *orfΔcoq2Δ* mutants had significantly reduced growth compared to *coq2Δ* (Fig. 6*A*) and nine *orfΔcoq2Δ* candidates had reduced growth when compared with the respective *orfΔ* single mutant (Fig. 6*B*). Additionally, seven *orfΔcoq2Δ* candidates had reduced growth when supplemented with CoQ_6_ as compared to supplementation with CoQ_2_, a hydrophilic CoQ isoform that can bypass trafficking defects (Fig. 6*C*). In total, seven autophagic candidate genes had significantly reduced growth in all three screens and thus are essential genes for the growth rescue mediated by exogenous CoQ_6_: *ATG6*, *ATG8*, *ATG9*, *ATG11*, *ATG12*, *ATG16*, and *PEP4*. Importantly, *ATG1*, *ATG2*, *ATG4*, *ATG7*, *ATG32* and *ATG36* are not essential for CoQ_6_ trafficking despite being essential for either autophagy or two types of selective autophagy. The finding that *ATG11* is required suggests that other selective autophagy pathways may play a role (62). This suggests that only a subset of the core autophagic machinery is required for CoQ_6_ uptake and trafficking. Interestingly, all seven *orfΔcoq2Δ* mutants that correspond to the seven autophagic genes identified as necessary for CoQ_6_ transport had similar exogenous CoQ_6_ content when compared with the *coq2Δ* mutant (Fig. 7*A*). This observation contrasts with the increased exogenous CoQ_6_ content in both the *nat3Δcoq2Δ* and *vps1Δcoq2Δ* mutants.

**Figure 6:**
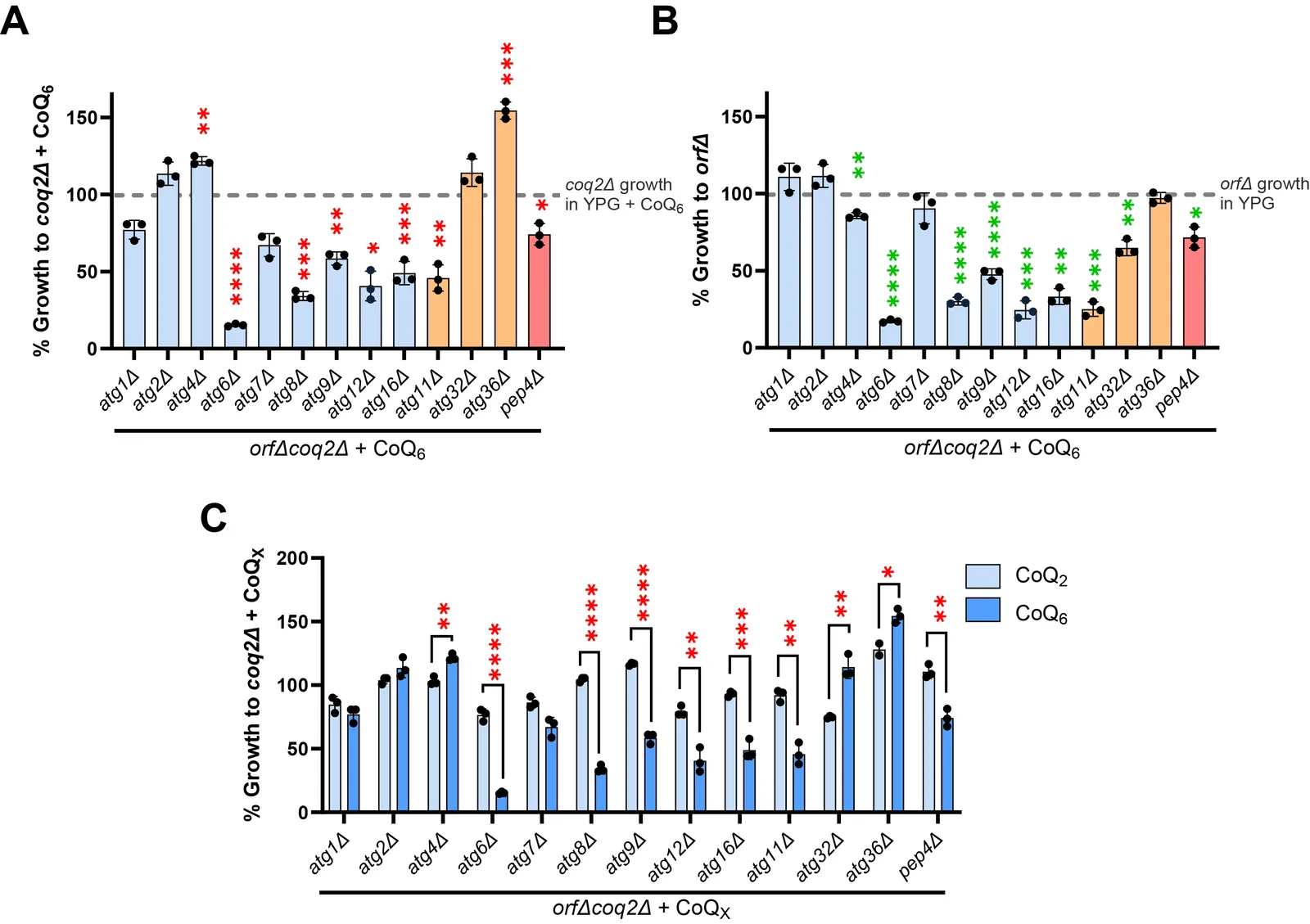
**Seven autophagic genes, *ATG6*, *ATG8*, *ATG9*, *ATG11*, *ATG12*, and *PEP4*, are essential for trafficking of exogenous CoQ_6_.** Mutants were grown in YPGG supplemented with or without exogenous CoQ_6_. The Abs_600nm_ was observed over seven days and the final growth measurement on day seven was compared to several controls. Columns represent the average degree of rescue (in %) ± standard deviation. *A*, the degree of rescue of the *orfΔcoq2Δ* mutants was compared to the *coq2Δ* + CoQ_6_. Of the thirteen candidates screened, seven mutants showed a significantly decreased ability to rescue growth in the presence of exogenous CoQ_6_. The *blue bars* represent mutants with disrupted autophagy, the *orange bars* represent mutants with disrupted selective autophagy, and the *red bar* represents the mutant with disrupted vacuolar turnover. *B*, since a single deletion can compromise the ability of a mutant to grow in YPG, the degree of rescue of the previous candidates was compared to the corresponding *orfΔ* single mutant. Nine *orfΔcoq2Δ* mutants had significantly decreased rescue with exogenous CoQ_6_ compared to growth of the respective *orfΔ* single mutant. *C*, the degree of rescue of *orfΔcoq2Δ* mutants with exogenous CoQ_2_ and CoQ_6_ was compared to the corresponding positive controls (*coq2Δ* + CoQ_2_ or *coq2Δ* + CoQ_6_) and is represented side by side. Seven candidates (*atg6Δ*, *atg8Δ*, *atg9Δ*, *atg11Δ*, *atg12Δ*, *atg16Δ*, and *pep4Δ*) had decreased growth rescue with CoQ_6_ compared to growth rescue with CoQ_2_ and compared to controls. The normalization variable is considered 100%. Asterisks denote the different degrees of significance as determined by unpaired t-tests between the *orfΔcoq2Δ* mutants with exogenous CoQ_6_ compared to the relevant control. ∗, *p* ≤ 0.05; ∗∗, *p* ≤ 0.01; ∗∗∗, *p* ≤ 0.001; and ∗∗∗∗, *p* ≤ 0.0001; ns, *p* > 0.05.

**Figure 7:**
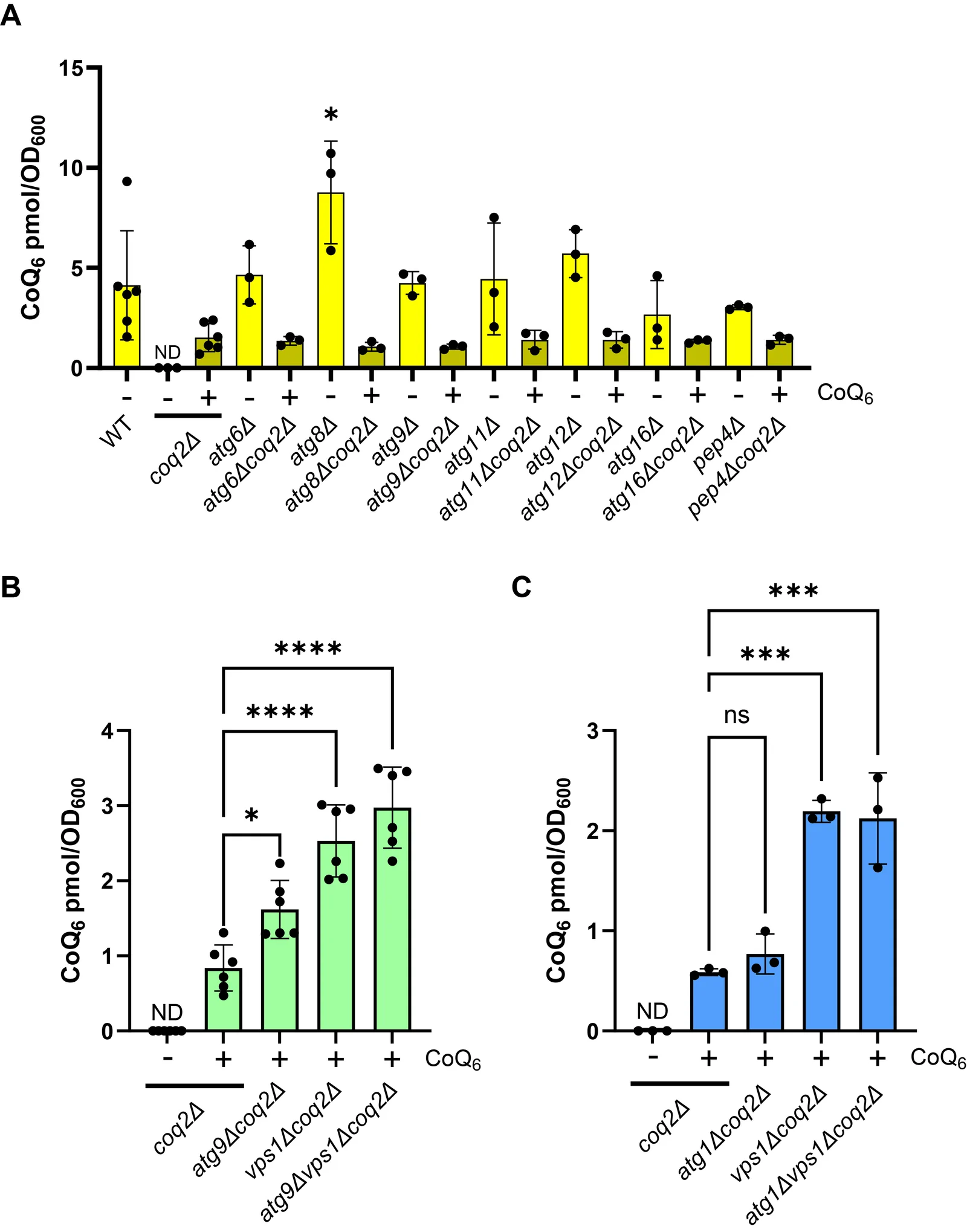
**Vps1-dependent CoQ_6_ accumulation is independent of *ATG* genes.** The cell-associated CoQ_6_ content was determined from lipid extracts of whole cells after three days of growth in YPD with or without 2 μM CoQ_6_. CoQ_6_ levels were determined using HPLC-MS. Three biological replicates were included for all candidates. *A*, the endogenously synthesized CoQ_6_ content of *orfΔ* mutants and the CoQ_6_ content from exogenous supplementation of *orfΔcoq2Δ* double mutants whose *ORF* is required for efficient CoQ_6_ trafficking is shown. *B*, the CoQ_6_ content from exogenous supplementation of the *atg9Δvps1Δcoq2Δ* is shown compared to controls. *C*, the CoQ_6_ content from exogenous supplementation of the *atg1Δvps1Δcoq2Δ* is shown compared to controls. Significance was determined using a one-way ANOVA with Dunnett’s multiple comparison test. ∗, *p* ≤ 0.05; ∗∗, *p* ≤ 0.01; ∗∗∗, *p* ≤ 0.001; and ∗∗∗∗, *p* ≤ 0.0001; ns, *p* > 0.05; ND indicates levels were below detection limit.

We next determined whether the accumulation of exogenous CoQ_6_ observed in the *vps1Δcoq2Δ* mutant would be affected by a complete block of autophagy. To test this, we prepared two triple mutants (*atg9Δvps1Δcoq2Δ* and *atg1Δvps1Δcoq2Δ*) in order to determine how these *ATG* gene deletions impacted the extent of uptake of exogenous CoQ_6_. While both Atg9 and Atg1 are required for autophagy, Atg9 is required for CoQ_6_ trafficking, while Atg1 is dispensable. We observed that the *atg9Δvps1Δcoq2Δ* triple mutant accumulated CoQ_6_ content similar to that of the *vps1Δcoq2Δ* double mutant (Fig. 7*B*). This result suggests that Vps1 acts upstream of Atg9, a transmembrane phospholipid scramblase that functions in both autophagy and in CoQ_6_ trafficking. We also examined the CoQ_6_ content accumulating in the *atg1Δvps1Δcoq2Δ* triple mutant. Although Atg1 is essential for autophagy, we found that the content of exogenously supplied CoQ_6_ in the *atg1Δvps1Δcoq2Δ* triple mutant was similar to that of the *vps1Δcoq2Δ* double mutant (Fig. 7*C*). This result indicates that deletion of *ATG1*, and its disruption of canonical autophagy, does not impact the CoQ_6_ trafficking pathway resulting from the absence of *VPS1*, and provides further support that these two processes are distinct.

### Two endomembrane trafficking genes are also essential for CoQ_6_ uptake and trafficking

Since only some of the autophagic genes that were screened are essential for CoQ_6_ uptake and trafficking, we postulate that the proteins encoded by these genes participate in a distinct endomembrane trafficking pathway utilized for uptake and trafficking of CoQ_6_. Therefore, we screened two additional endomembrane trafficking genes, *VPS4* and *VPS38*, using the *orfΔcoq2Δ* model system. *VPS4* encodes an ATPase that facilitates subunit exchange in complex III of the endosomal sorting complex for protein transport pathway (ESCRT-III) for effective membrane remodeling in the multivesicular body (58). *VPS38* encodes a protein subunit of the PtdIns 3-kinase complex II, which is involved in endomembrane trafficking but whose activity is dispensable for autophagy (59, 60). The *vps4Δcoq2Δ* and *vps38Δcoq2Δ* double mutants had reduced growth in YPGG with exogenous CoQ_6_ when compared with both *coq2Δ* cultured with exogenous CoQ_6_ (Fig. 8*A*) and the respective *orfΔ* single mutant (Fig. 8*B*). Additionally, both double mutants also had reduced growth in YPGG supplemented with CoQ_6_ as compared to supplementation with exogenous CoQ_2_ (Fig. 8*C*). The decreased growth of the *vps4Δcoq2Δ* and *vps38Δcoq2Δ* mutants in each of these three comparisons indicates that both *VPS4* and *VPS38* are essential genes for CoQ_6_ uptake and trafficking. Surprisingly, the *vps4Δ* single mutant had remarkably high content of CoQ_6_ (Fig. 8*D*). However, unlike the *vps1Δcoq2Δ* mutant, which accumulated high amounts of exogenous CoQ_6_, the *vps4Δcoq2Δ* and *vps38Δcoq2Δ* double mutants cultured in YPD medium supplemented with exogenous CoQ_6_ had similar whole-cell CoQ_6_ content when compared with the *coq2Δ* mutant (Fig. 8*D*). In conclusion, *VPS4* and *VPS38* are essential for uptake and trafficking of exogenous CoQ_6_ and implicate a novel endomembrane trafficking pathway that requires a subset of autophagic proteins.

**Figure 8:**
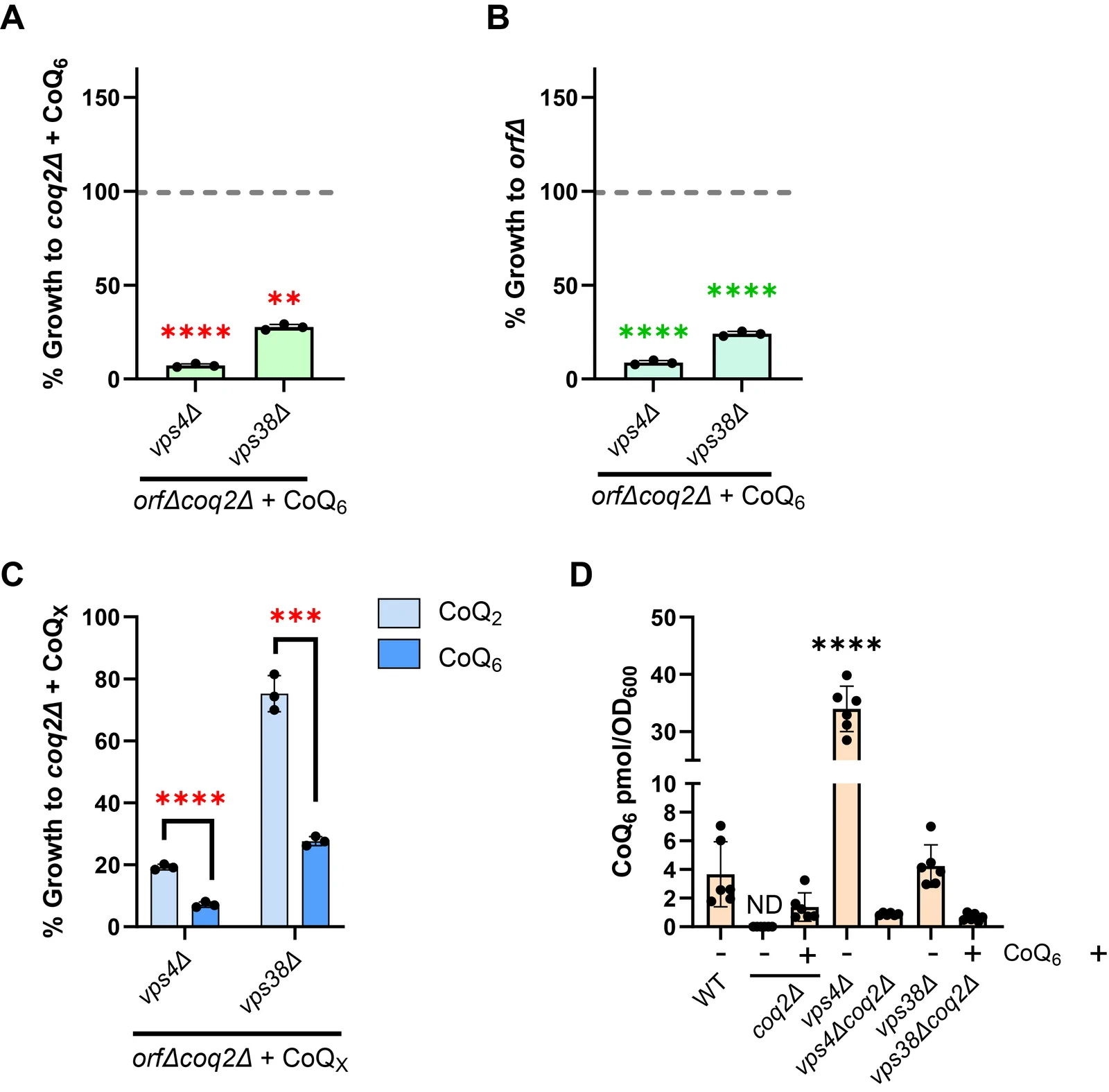
***VPS4* and *VPS38* are essential for trafficking of exogenous CoQ_6_.** Mutants were grown for seven days in YPGG supplemented with or without exogenous CoQ_6_. The Abs_600nm_ after seven days of growth was compared to several controls, as described in Figure 7. Columns represent the average degree of rescue (in %) ± standard deviation. *A*, the degree of rescue of the *orfΔcoq2Δ* mutants was compared to the *coq2Δ* + CoQ_6_. *B*, the degree of rescue of the *orfΔcoq2Δ* mutants was compared to the corresponding *orfΔ* single mutant. *C*, the degree of rescue of *orfΔcoq2Δ* mutants with exogenous CoQ_2_ and CoQ_6_ was compared to the corresponding positive controls (*coq2Δ* + CoQ_2_ or *coq2Δ* + CoQ_6_) and is represented side by side. Asterisks in *panels* (*A–C*) denote the different degrees of significance as determined by unpaired t-tests between the *orfΔcoq2Δ* mutants with exogenous CoQ_6_ compared to the relevant control. *D*, the cell-associated CoQ_6_ content was determined from lipid extracts of whole cells after three days of growth in YPD with or without 2 μM CoQ_6_. CoQ_6_ levels were determined using HPLC-MS. Three biological replicates were included for all candidates. Significance for *panel D* was determined using a one-way ANOVA with Dunnett’s multiple comparison test. *Black asterisks* denote significance of *orfΔ* mutants compared to WT, *green asterisks* denote significance of *orfΔcoq2Δ* double mutants compared to respective *orfΔ* control, and *red asterisks* denote significance compared to *coq2Δ* with exogenous CoQ_6_. ∗, *p* ≤ 0.05; ∗∗, *p* ≤ 0.01; ∗∗∗, *p* ≤ 0.001; and ∗∗∗∗, *p* ≤ 0.0001; ns, *p* > 0.05; ND indicates levels were below detection limit.

## Discussion

Here we extended the use of the *orfΔcoq2Δ* yeast model system to identify nine essential genes for trafficking of exogenously supplied CoQ_6_: *ATG6*, *ATG8*, *ATG9*, *ATG11*, *ATG12*, *ATG16*, *PEP4*, *VPS4*, and *VPS38*. The *coq2Δ* single mutant is able to assimilate exogenous CoQ_6_ and the CoQ_6_ content does not significantly change over time (Fig. S1). These data suggest that the *coq2Δ* mutant can regulate its whole-cell CoQ_6_ content. In contrast, the double *orfΔcoq2Δ* mutants we identified failed to traffic exogenous CoQ_6_ to respiratory complexes despite the presence of CoQ_6_ being cell associated. Curiously, the *vps1Δcoq2Δ* mutant accumulated a very high CoQ_6_ content (Fig. 1), even though it failed to restore growth in YPG, suggesting CoQ_6_ is mislocalized outside of mitochondrial respiratory complexes. We propose a model in which Vps1 and selective autophagic and endocytic machinery play a central role in a previously uncharacterized endomembrane trafficking pathway to deliver exogenous CoQ_6_ to mitochondrial respiratory complexes (Fig. 9). We postulate that exogenous CoQ_6_ initially assimilates into the plasma membrane before undergoing endocytosis. The resulting endocytic vesicles are subsequently sorted through the multivesicular body *via* ESCRT machinery and phosphorylation of PtdIns. Our work suggests that exogenous CoQ_6_ may be delivered to the vacuole, and that vacuolar CoQ_6_ is finally transported to mitochondria, possibly *via* membrane contact sites. Future studies are required to further characterize and identify additional components of this pathway.

**Figure 9:**
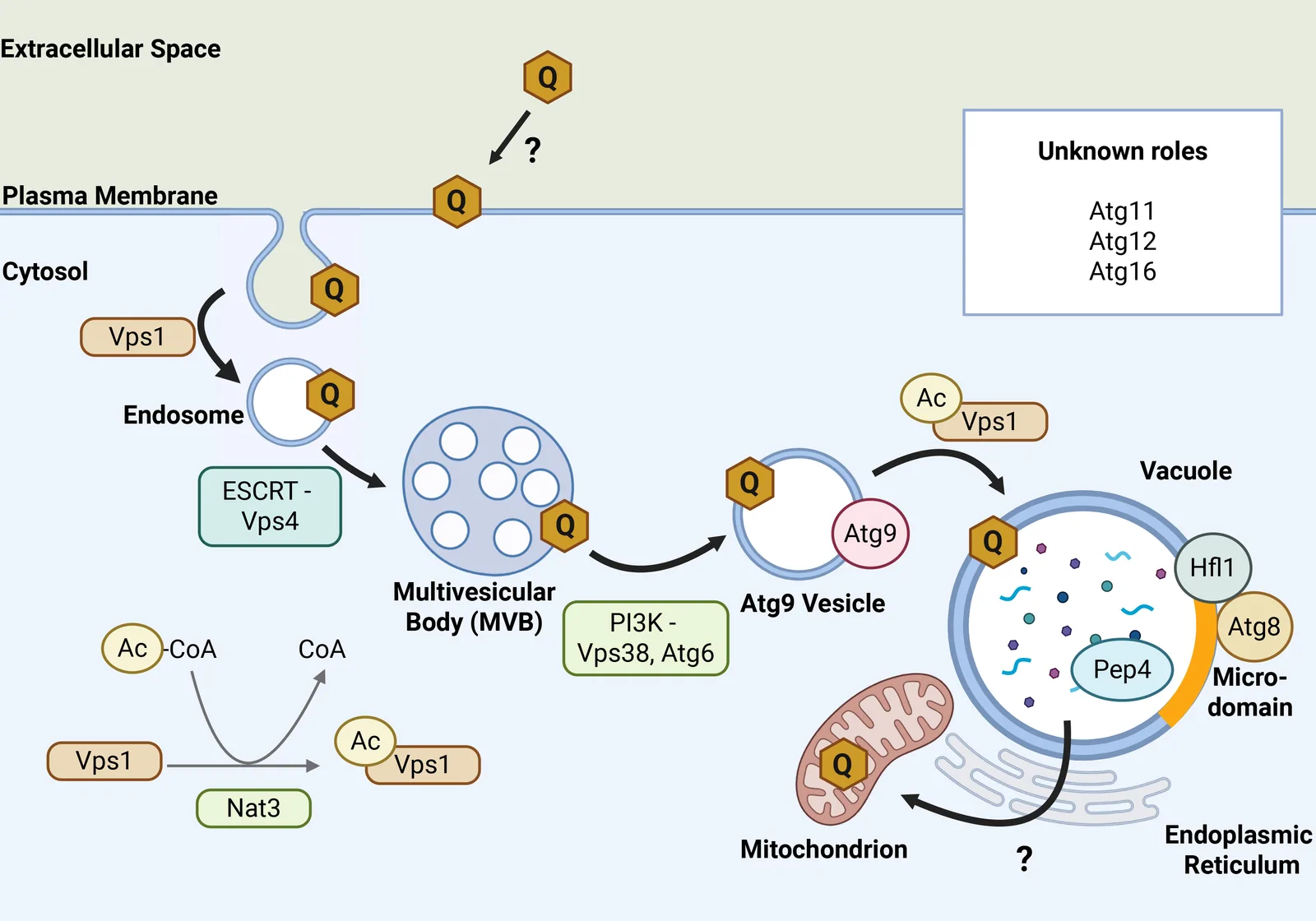
**Scheme of proposed CoQ_6_ uptake pathway.** Vps1 and endocytic machinery facilitate the uptake of exogenous CoQ, likely solubilized by peptide components in the medium, and assimilated directly into the plasma membrane. Endosomes containing CoQ are trafficked to the multivesicular body. CoQ is sorted into Atg9-containing vesicles *via* ESCRT machinery and the Vps38 PI3K complex. Atg9-containing vesicles are trafficked to the vacuole *via* Vps1. The vacuolar microdomain is depicted in *yellow*, and is known to depend on the interaction of Atg8 with Hfl1. Cargo from the vesicle is then degraded by vacuolar hydrolases and peptidases. CoQ is then delivered to mitochondria through unknown mechanisms, possibly through membrane contact sites. Created in BioRender.

Disruption of Vps1 has been shown to profoundly disrupt endomembrane trafficking. Upon deletion of *VPS1*, direct vesicular trafficking between the Golgi and vacuole is disrupted so that cargo from the Golgi is first diverted to the plasma membrane and then delivered to the vacuole *via* endocytosis (63, 64). Moreover, Vps1 also has a direct role in regulating vacuolar morphology. Under normal growth conditions, wild-type yeast have one large vacuole which may be multilobed or contain invaginations, while the *vps1Δ* mutant has many small vacuole fragments grouped around a larger, smooth vacuole (65). We postulate that the accumulation of exogenous CoQ_6_ in the *vps1Δcoq2Δ* mutant is caused by increased trafficking through the plasma membrane and an accumulation of endocytic vesicles that are not delivered to the vacuole. Exogenous CoQ_6_ content is increased in subcellular fractions enriched in plasma membranes and in fractions containing mitochondria and other intracellular membranes in the *vps1Δcoq2Δ* mutant as compared to the *coq2Δ* mutant (Fig. 2). This finding is consistent with a defect in trafficking and an aberrant location of CoQ_6_.

We identified three *vps1* point mutants that fail to effectively deliver exogenous CoQ_6_ to respiratory complexes. Although the *vps1-T63A* and *vps1-I649K* mutants were reported to have opposing effects on endocytosis and endomembrane trafficking (45), expression of either mutant failed to restore growth of the *vps1Δcoq2Δ* mutant and led to the accumulation of high cellular content of exogenous CoQ_6_ (Fig. 3). Vps1 has been shown to deliver Atg9, a lipid scramblase and the only transmembrane protein in the core autophagic machinery, to the phagophore assembly site (PAS). Interestingly, expression of *vps1-*T63A and *vps1-*I649K in an autophagy-deficient *atg1Δvps1Δ* double mutant increased the number of Atg9-GFP puncta outside the PAS (34). Before trafficking to the PAS, Atg9 is found in single-membrane, Golgi-derived vesicles sometimes referred to as Atg9 reservoirs (66). It is possible that Atg9 has a moonlighting role in vesicular transport of CoQ_6_ and defects in trafficking of Atg9 to the PAS also result in a trafficking defect of CoQ_6_. Expression of *vps1-D2P* failed to restore growth of the *vps1Δcoq2Δ* double mutant but resulted in exogenous CoQ_6_ content comparable to that of the *coq2Δ* mutant (Fig. 4). The *vps1-D2P* mutant has been shown to be unable to fuse autophagosomes with the vacuole, resulting in an accumulation of autophagosomes within the cytosol (35). However, the trafficking of Atg9 to the PAS in the *vps1-*D2P mutant was not investigated in this study. Together, these findings suggest that Vps1 might play multiple roles in CoQ_6_ trafficking and suggest a potential link between Vps1-dependent Atg9 transport and CoQ_6_ trafficking.

Expression of constitutively acetylated *vps1-D2M* in the *nat3Δcoq2Δ* mutant was unable to rescue growth in YPG medium with exogenous CoQ_6_, demonstrating that acetylation of other proteins by Nat3 is required for CoQ_6_ uptake and trafficking (Fig. 5). Nat3 acetylation of other proteins besides Vps1 is also required for efficient autophagy; specifically, deletion of *NAT3* resulted in decreased localization of Atg9 at the PAS (35). We observed an accumulation of exogenous CoQ_6_ in the *nat3Δcoq2Δ* mutant, further suggesting a connection between Atg9 transport and CoQ_6_ trafficking. Although the biological roles and mechanisms of N-terminal acetylation are not fully elucidated, a growing body of work shows that N-terminal acetylation of the target substrate can be used for protein folding, protein degradation, subcellular localization, and complex formation (67, 68).

Notably, several of the core autophagic genes screened were not required for CoQ_6_ transport, indicating that autophagy is dispensable for the uptake and trafficking of exogenous CoQ_6_ (Fig. 6). Interestingly, autophagic *orfΔcoq2Δ* mutants whose *ORF* corresponds to an essential CoQ-trafficking gene had similar CoQ_6_ content when compared with *coq2Δ* (Fig. 7*A*). This is in contrast to the accumulation of CoQ_6_ observed in the *vps1Δcoq2Δ* and *nat3Δcoq2Δ* mutants (Fig. 5). Analyses of the *atg1Δvps1Δcoq2Δ* and *atg9Δvps1Δcoq2Δ* triple mutants revealed that neither deletion of *ATG1* nor *ATG9* impacted the high content of CoQ_6_ that accumulated in the *vps1Δcoq2Δ* genetic background (Fig. 7). This result indicates that the process of canonical autophagy is distinct from the trafficking of CoQ_6_, and that Vps1 functions upstream of Atg9 in the CoQ-trafficking pathway.

The study of autophagy continues to expand, revealing an increasing number of non-canonical functions for the autophagic machinery. Many of these non-canonical roles involve degradative and secretory pathways that rely on Atg8 or its mammalian homolog LC3 (41, 42, 43, 44). One such example is the secretion of yeast acyl-coenzyme A binding protein (Acb1) *via* an unconventional protein secretion (CUPS) pathway, which relies on machinery strikingly similar to that of exogenous CoQ_6_ uptake and trafficking (69, 70, 71). The CUPS pathway includes Atg8 and Atg9, membrane modification *via* the PI3K complex, and sorting through the multivesicular body. Atg9 is a lipid scramblase involved in multiple autophagic steps, and its mammalian homologs show other non-canonical functions that are widespread throughout the cell, such as actin organization, lysosome maintenance, lipid-droplet biogenesis, immune signaling, and plasma membrane repair (72). However, CUPS-mediated secretion requires Atg7 (73), and since Atg7 is not essential for CoQ trafficking, the involvement of CUPS is questionable.

It is possible that the PI3K complex phosphorylates PtdIns of CoQ_6_-containing vesicles, but other lipid requirements for effective trafficking are not known. These CoQ_6_-containing vesicles are non-autophagic but likely contain Atg8 and Atg9. Atg12 and Atg16 act as E3-like conjugating proteins for the lipidation of Atg8 to phosphatidylethanolamine and are essential for CoQ_6_ uptake and trafficking. However, Atg7, an E1-like conjugating enzyme, and Atg4, a cysteine protease that cleaves the C-terminus of Atg8, are dispensable for CoQ_6_ trafficking. Moreover, the requirement of Pep4 for efficient CoQ_6_ uptake and trafficking indicates that these vesicles must be processed within the vacuole. It seems plausible that CoQ_6_ is possibly handed off through the endoplasmic reticulum *via* membrane contact sites, such as the Endoplasmic Reticulum-Mitochondria Encounter Structure (ERMES) (11, 12, 13).

We also identified two endomembrane trafficking genes, *VPS4* and *VPS38*, as essential for uptake and trafficking of exogenous CoQ_6_ (Fig. 8). Both genes are dispensable for autophagy but play critical roles in endomembrane trafficking, further supporting an essential role for vesicular trafficking in endogenous CoQ_6_ transport. This agrees with previous work showing functional endomembrane trafficking is essential for normal CoQ distribution (32). Unexpectedly, the CoQ_6_ content of the *vps4Δ* mutant was nearly ten-fold higher than that of the wild-type (Fig. 8). This increase in CoQ_6_ content might be connected to other investigations that have identified a role for Vps4 in lipid droplet consumption and vacuole homeostasis (74, 75). Perhaps CoQ_6_ accumulates with decreased turnover of other neutral lipids from lipid droplets and merits future investigation.

We speculate that CoQ_6_ transport to the vacuolar membrane may precede its transfer to mitochondria. Surprisingly, several of the genes essential for CoQ_6_ trafficking also play a role in the formation of liquid-ordered microdomains (L_o_) at the vacuolar membrane, including *VPS4* (76); *PEP4*, *ATG6*, and *ATG8* (77). L_o_ membrane domains are sterol-enriched regions of the vacuole whose function is necessary for uptake of neutral lipids (78, 79). Vacuolar microdomains have been shown to participate in microautophagy of lipid droplets (microlipophagy), a process which occurs during stationary phase and carbon starvation, and can be independent of canonical autophagy (80). It is particularly noteworthy that the function of Atg8 in establishing the vacuolar microdomains is independent of its processing by Atg4 and its lipidation by Atg7 (81). This is compatible with our finding that Atg8 is needed for trafficking of CoQ_6_, and is independent of both Atg4 and Atg7. Instead, unlipidated Atg8 is anchored by Hfl1 at the vacuole membrane (82), an association required for the formation of the L_o_ domains. Expansion of the L_o_ domains in the vacuole membrane appears to be indispensable for the engulfment of cargo, a process that also depends on the yeast Niemann-Pick homologs, Ncr1 and Ncr2 (78). Importantly, mammalian NPC1 has been shown to mediate transport of exogenous CoQ in mammalian cells (83).

This work corroborates findings in mammalian cell models that CoQ uptake occurs *via* the endomembrane system (31) and that the lytic compartment acts as a central hub of CoQ distribution (83, 84). Uptake in mammalian cells is mediated by the CD36 receptor protein and its subsequent transport to mitochondria depends on NPC1-mediated transport to the lysosome, which precedes its ultimate delivery to the mitochondria (28, 83, 85, 86). STARD7 was identified as an intracellular CoQ transfer protein required for ferroptosis suppression (87). The degree to which endogenous CoQ is trafficked from mitochondria to the plasma membrane is regulated in response to the isoprene biosynthetic pathway (88). Conversely, defects in CoQ biosynthesis alter cholesterol homeostasis in neurons (89). However, a yeast cytosolic CoQ-binding transfer protein has yet to be discovered (30, 90). More work is needed to determine how the biosynthesis and transport of sterol and nonsterol products are balanced.

This study has several limitations that warrant further investigation. Our assay of CoQ_6_ uptake and trafficking to mitochondria depends on the restoration of mitochondrial respiratory chain function as assayed by growth on glycerol. Thus, genes required for respiration (such as *ATG14*) cannot be interrogated by this method. Furthermore, genomic redundancies in components that function in trafficking pathways will obscure their identification, and compensatory changes can be elicited in response to gene deletions. For example, mitochondria depend on membrane contact sites for phospholipid transport, and this requirement is satisfied and compensated for by the presence of either ERMES or the vacuole and mitochondrial patch (vCLAMP); however, the absence of both is lethal (91). We note that mutations in *vps1* and the select endocytic and autophagy genes may have ripple effects in membrane trafficking that affect normal vesicular trafficking pathways. Finally, it would be quite informative to be able to “track” the location of exogenously added CoQ_6_ as this would help identify the cellular compartment where it accumulates. Recently a CoQ-Azide probe was developed that enabled the visualization of CoQ *via* fluorescence-based imaging (83). It would be fascinating to extend this approach to the yeast model to identify the highly conserved eukaryote players, pathways and compartments that mediate CoQ trafficking.

In summary, we have shown that Vps1 and select components of the core autophagic machinery play a non-canonical role in the uptake and trafficking of exogenous CoQ_6_ to mitochondria. Our results suggest that uptake and intracellular transport of CoQ occur through a novel pathway that relies on select autophagic and endomembrane trafficking proteins and relies on the lytic compartment as a transport hub.

## Experimental procedures

All reagents were obtained commercially from Fisher Scientific unless noted otherwise.

### Coenzyme Q isoforms

CoQ_2_ was obtained from Cayman Chemical, CoQ_4_ was obtained from Sigma-Aldrich, and CoQ_6_ from Avanti. Each was dissolved in ethanol, and the concentration was calculated using the Abs_275 nm_ in a 1 ml UV cuvette and the corresponding molar extinction coefficients (13,700 M^−1^ cm^−1^ for CoQ_2_; 14,200 M^−1^ cm^−1^ for CoQ_4_; 14,700 M^−1^ cm^−1^ for CoQ_6_) (92).

### Yeast strains, growth media, and verification of mutants

*S. cerevisiae* strains used in this study were derived from S288C (93). Yeast strains used in this study are described in Table S1. Standard growth media for yeast was prepared as described (94) and included 1% Bacto yeast extract (Gibco), 2% Bacto peptone (Gibco), and 2% dextrose (YPD) or 3% glycerol (YPG). YPG medium supplemented with 0.5% dextrose (YPGG) was used for exogenous CoQ_6_ rescue assays (see below). Solid plate medium contains an additional 2% Bacto agar.

To select for mutants that contain a *KanMX* cassette or a *NatMX6* cassette, YPD + 200 mg/L G418 (Gibco) or YPD + 100 μg/ml Nourseothricin (Thermo Scientific Chemicals) plates were used, respectively. To select for mutants with a *LEU2* or *URA3* cassette, synthetic dextrose media without leucine or uracil (SD –Leu or SD –Ura) (2% dextrose, amino acid supplement lacking leucine or uracil, 0.17% yeast nitrogen base without amino acids and ammonium sulfate (Difco), 0.5% ammonium sulfate, 0.14% monosodium phosphate) plates were used. For plasmid selection (see below) SD –Ura was used.

The *rho* status of the cells was also confirmed using JM6 and JM8 as *rho*^0^ test strains (95). Additionally, prior to the initiation of CoQ_6_ rescue experiments, we confirmed the ability of *orfΔ* mutants to grow on YPG to ensure that recovery of respiration of the corresponding *orfΔcoq2Δ* double mutant with exogenous CoQ_6_ is possible.

### Generation of double and triple deletion mutants

*orfΔcoq2Δ* mutants, except for the *vps1Δcoq2Δ* and the *nat3Δcoq2Δ*mutant, were created *de novo* using a PCR-based gene deletion strategy (96). In all cases, *COQ2* was replaced with the *LEU2* cassette or *NatMX6* cassette. The *LEU2* cassette was amplified from the pRS305 vector (Table S2) using primers to which homologous sequences to the upstream of the *COQ2* start codon and downstream of the *COQ2* stop codon were included. The *COQ2::natMX6* disruption cassette was amplified from *vps1Δcoq2Δ* genomic DNA using *COQ2* upstream and downstream primers. The *atg1Δvps1Δcoq2Δ* and *atg9Δvps1Δcoq2Δ* triple mutants were created *de novo* by knocking out *ATG1* and *ATG9* using a *URA3* cassette in the *vps1Δcoq2Δ* background. Details about these homologous sequences are described in Table S3. Following PCR clean up (Thermo Fisher Scientific), the PCR products were used to transform an *orfΔ* mutant strain using the lithium acetate/single-stranded carrier DNA/polyethylene glycol method (97). Transformed cells were selected twice on SD –Leu, SD –Ura, or YPD + 100 μg/ml Nourseothricin plates. Genomic DNA isolation followed by PCR was used to verify the correct integration of the disruption cassette at the correct gene locus. In these verification steps, primers upstream to the start codon or downstream of the stop codon of the marker were used with primers within the disruption cassette to verify that the marker was integrated at the correct locus.

### Plasmid generation and transformation

Plasmids used in this study are listed in Table S2. To generate pKA677-D2M and pKA677-D2P, mutations were made to pKA677 using the QuikChange Lightning site-directed mutagenesis kit (Agilent). Yeast cells were transformed with plasmid using the lithium acetate/single-stranded carrier DNA/polyethylene glycol method (97). Transformed cells were selected twice on SD –Leu or SD –Ura plates.

### Exogenous CoQ rescue assays

To identify genes that are essential for efficient uptake and trafficking of exogenous CoQ_6_, or to determine if plasmid expression can restore trafficking of exogenous CoQ_6_, the exogenous CoQ rescue assay was used. Yeast mutants were grown overnight in YPD, or SD –Ura if transformed with a plasmid, at 30 °C, 250 rpm. The next day, the OD_600_ was measured by taking the absorbance at 600 nm of 1 ml of cell culture or appropriate dilution in water, and yeast strains were sub-inoculated into tubes containing 5 ml of YPGG supplemented with CoQ (2 μM of CoQ_6_ or 100 μM of CoQ_2_) (29) or ethanol at a cell density of 0.2 OD_600_. In all experiments, the amount of ethanol added to the culture (containing CoQ or as a vehicle) was 1% of the total culture volume. Yeast were allowed to grow at 30 °C, 250 rpm for 7 days, and the culture density was measured periodically by taking the absorbance at 600 nm (Abs_600 nm_) of a 200 μl aliquot of cell culture or appropriate dilution in water in order to construct growth curves. CoQ_6_ rescue is always represented as a percentage with designated control, considered at 100% and represented as a dashed line in the graphics (see examples in Fig. 7). Abs_600 nm_ from the last time point, approximately 168 h of growth, was used to calculate the degree of rescue. To construct the growth curves in Figure 1 and Figure 2, the OD_600_ was taken.

### Determination of CoQ_6_

Lipids were extracted for analysis from an aliquot of the cell cultures equivalent to 5 to 10 OD_600_. For strains with little growth, cells equivalent to 5 to 10 OD_600_ were pelleted from large cultures. Collected cell pellets were washed twice in 1X phosphate-buffered saline (1X-PBS) (137 mM sodium chloride, 2.7 mM potassium chloride, 10 mM disodium phosphate, 1.8 mM monopotassium phosphate, pH = 7.4). Lipid extractions and the following analysis of CoQ content were performed as previously described (98). The cell pellet was resuspended in water and an aliquot was transferred into 2 ml methanol and cells were lysed in the presence of glass beads. Lipids were extracted twice in the presence of the internal standard CoQ_4_ (Sigma-Aldrich) with the addition of 2 ml petroleum ether followed by vortexing each time. Lipids were dried under nitrogen gas and reconstituted into 200 μl of 1 mg/ml benzoquinone in ethanol prior to analysis. A set of standards was prepared at the same time as the samples by extracting known amounts of CoQ_6_ and CoQ_4_ and used to generate a standard curve with a simple linear fit forced through the origin. For the assays presented, the limit of detection (LOD) and limit of quantification (LOQ) of CoQ_6_ were 0.03 and 0.06 pmol, respectively, and all samples in which CoQ_6_ was detected contained >0.3 pmol of CoQ_6_ and thus well above the LOQ. CoQ_6_ concentrations less than 0.03 pmol is below the lowest concentration of the standard curve and reported as not detectable (ND). The total CoQ_6_ content was normalized against OD_600_ of extracted cells.

### Spot plate assays

To determine the viability of strains on plated medium, growth was analyzed using spot plate assays. Cells were grown at 30 °C, 250 RPM and the incubation time and medium is stated in the appropriate figure caption. The OD_600_ of cultured cells was measured and cells were then diluted to an OD_600_ = 0.2 in sterile water. A series of 5-fold dilutions were prepared. 2 μl of each dilution was plated onto YPD and YPG plates. Dilutions were also plated onto SD –Ura if cells were transformed with pRSQ2. Plates were incubated at 30 °C for 3 to 4 days. Images were captured using Azure 300 Imager (Azure Biosystems) at 472 nm with no emission filter.

### Immunoblot analysis

Sample preparation was adapted from a lithium acetate/sodium hydroxide method (99). The cells were pelleted and resuspended in 300 μl of 2 M lithium acetate and incubated on ice for 5 min. Samples were centrifuged and pellets were resuspended in 300 μl of 0.4 M sodium hydroxide, then incubated on ice for 5 min. After this step, a 10 μl aliquot of each sample was saved for protein quantification. Samples were resuspended in 1X SDS sample buffer (50 mM Tris pH 6.8, 10% glycerol, 2% SDS, 0.1% bromophenol blue, and 1.33% β-mercaptoethanol) to a final protein concentration of 2 mg/ml and boiled for 5 min. Samples were stored at −20 °C until further use. 25 μg of protein was loaded in individual lanes and separated by SDS gel electrophoresis on 12% Tris-glycine polyacrylamide gels. Proteins were subsequently transferred to 0.45 μm Immobilon-P membrane (Millipore). Blots were stained with Ponceau S dye for visualization of protein lanes and assessment of equal protein loading. After blocking (5% milk and 0.1% Tween-20 in 1X-PBS), blots were incubated with primary and secondary antibodies described in Table S4. Immunoblot images were visualized with a LICOR Odyssey Infrared scanner (LICORbio), and relative protein levels were quantified by band densitometry using ImageJ (100).

### Complementation of *orfΔcoq2Δ* mutants with yeast *COQ2*

To re-introduce *COQ2* in the *orfΔcoq2Δ* double mutants, *orfΔcoq2Δ* and *coq2Δ* mutants were transformed with pRS316 or pRSQ2 as described previously (29). WT and the corresponding single *orfΔ* mutants were transformed with pRS316 to serve as controls. Cells containing the plasmid were double selected on SD –Ura plates. The ability of *orfΔcoq2Δ* mutants to recover their respiratory capacity with pRSQ2 was analyzed using spot plate assays.

### GFP-Atg8 cleavage assay

The autophagy phenotype of yeast strains was observed using the GFP-Atg8 cleavage assay (54, 55). Cells were transformed with pGFP-Atg8, which expresses a recombinant GFP-Atg8 fusion protein under the endogenous Atg8 promoter and grown overnight in SD –Ura liquid medium to an OD_600_ of ∼1.2. Approximately 25 ml of culture was pelleted and washed once with sterile water. Cells were resuspended in nitrogen starvation medium (SD –N) (0.17% yeast nitrogenase base without amino acids or ammonium sulfate, 2% dextrose, pH 6.0) and grown for six hours at 30 °C, 250 rpm. Cells were then pelleted and used for immunoblot analysis. Relative levels of GFP-Atg8 and cleaved GFP were quantified by band densitometry using ImageJ. Cleaved GFP band density was normalized against total GFP bands (both GFP-Atg8 and cleaved GFP) and results are reported relative to the *coq2Δ* control, considered at 100% and represented as a dashed line in the graphics.

### Yeast subcellular fractionation and purification of plasma membrane fractions

To isolate plasma membrane-enriched membrane fractions of cell lysates using density gradient centrifugation, we adapted published methods (101, 102). In brief, six OD_600 nm_ units of cells from a saturated overnight culture were added to 30 ml of YPD containing either 1% (vol/vol) ethanol (Sigma-Aldrich, catalog number 459836-1L) or 2 μM CoQ_6_ and grown in a shaker at 30 °C for 18 h. Fifty OD_600 nm_ of culture was withdrawn and washed twice with sterile water. The cells were resuspended in 1 ml ice-cold lysis buffer (50 mM TRIS pH7.4 (Thermo Fisher Scientific, catalog number J60202.K2), 1 mM EDTA (Thermo Fisher Scientific, catalog number J15694.AP), 1 mM PMSF (Sigma-Aldrich, catalog number P7626-5G), proteinase inhibitor tablet (Roche, mini EDTA-free protease inhibitor cocktail, catalog number 11836170001), transferred to 2 ml screw-cap tube, and washed once with ice-cold lysis buffer. The cells were resuspended in 300 μl lysis buffer, 300 μl of glass beads (Sigma-Aldrich, catalog number G8772-500G) were added, and the suspension was vortexed seven times for 45 s followed by a 2-min pause on ice. After adding 350 μl lysis buffer, and briefly vortexing, the samples were centrifuged at 400*g* for 5 min at 4 °C, and the supernatant was transferred to a new microcentrifuge tube and centrifuged again. The supernatant was transferred to a new tube and 450 μl was mixed with 450 μl 76% renografin (660 mg/ml meglumine diatrizoate (Sigma-Aldrich, catalog number M5266-100G), 100 mg/ml sodium diatrizoate (Sigma-Aldrich, catalog number S4506), 50 mM TRIS pH 7.4, 1 mM EDTA). To make the density gradient, 800 μl of lysate was placed at the bottom of an ultracentrifuge tube and overlayed with four 800 μl layers of renografin at 34%, 30%, 26% and 22% respectively. The samples were centrifuged at 40,000 rpm with no break for 18-22 h at 4 °C in a SW55Ti rotor. Thirteen fractions of 285 μl each were taken from the top to the bottom of the ultracentrifuge tube. Fractions 9 to 11 contains most of the plasma membrane, while fractions 4 to 6 contained most of other cellular membranes. To pellet the membranes, the pooled fractions were mixed with 4 ml of ice-cold water and centrifuged at 100,000*g* for 1h at 4 °C. The pellets were resuspended in 100 μl of ice-cold lysis buffer. Plasma membrane extraction quality was checked by immunoblotting.

### Statistical analysis

Data shown in this work represent the mean ± standard deviation (SD) from at least three biological replicates. Statistical analyses and graphics were performed with GraphPad Prism 10 (GraphPad Software Inc). When a mutant is compared to WT or to a designated normalization variable (such as *coq2Δ* + CoQ_6_ or *orfΔ* mutant) an unpaired *t* test analysis was performed. Statistical analyses were performed with parametric one-way analysis of variance (ANOVA) correcting for multiple comparisons. Tukey’s test was used to compare all possible group means and Dunnett’s test was used to compare multiple groups against a single control group. The statistical significance is represented by the following: ∗, *p* ≤ 0.05; ∗∗, *p* ≤ 0.01; ∗∗∗, *p* ≤ 0.001; and ∗∗∗∗, *p* ≤ 0.0001; ns, *p* > 0.05. The denotation NS indicates values with “not significant” differences from the corresponding control.

## Data availability

This article contains supporting information. All study data are included in the article and/or supporting information.

## Supporting information

This article contains supporting information (29, 34, 45, 46, 47, 48, 49, 50, 93, 103, 104, 105, 106).

## Conflict of interest

The authors declare that they have no conflicts of interest with the contents of this article.

## References

[1] Hayashi K., Ogiyama Y., Yokomi K., Nakagawa T., Kaino T., Kawamukai M. (2014). Functional conservation of coenzyme Q biosynthetic genes among yeasts, plants, and humans. PLoS One.

[2] Wang Y., Hekimi S. (2013). Molecular genetics of ubiquinone biosynthesis in animals. Crit. Rev. Biochem. Mol. Biol..

[3] Alcazar-Fabra M., Navas P., Brea-Calvo G. (2016). Coenzyme Q biosynthesis and its role in the respiratory chain structure. Biochim. Biophys. Acta.

[4] Vercellino I., Sazanov L.A. (2022). The assembly, regulation and function of the mitochondrial respiratory chain. Nat. Rev. Mol. Cell Biol..

[5] Banerjee R., Purhonen J., Kallijarvi J. (2022). The mitochondrial coenzyme Q junction and complex III: biochemistry and pathophysiology. FEBS J..

[6] Guerra R.M., Pagliarini D.J. (2023). Coenzyme Q biochemistry and biosynthesis. Trends Biochem. Sci..

[7] Awad A.M., Bradley M.C., Fernandez-Del-Rio L., Nag A., Tsui H.S., Clarke C.F. (2018). Coenzyme Q10 deficiencies: pathways in yeast and humans. Essays Biochem..

[8] Stefely J.A., Pagliarini D.J. (2017). Biochemistry of mitochondrial coenzyme Q biosynthesis. Trends Biochem. Sci..

[9] Kemmerer Z.A., Robinson K.P., Schmitz J.M., Manicki M., Paulson B.R., Jochem A. (2021). UbiB proteins regulate cellular CoQ distribution in *Saccharomyces cerevisiae*. Nat. Commun..

[10] Khosravi S., Chelius X., Unger A.K., Rieger D., Frickel J., Sachsenheimer T. (2023). The UbiB family member Cqd1 forms a novel membrane contact site in mitochondria. J. Cell Sci..

[11] Eisenberg-Bord M., Tsui H.S., Antunes D., Fernandez-Del-Rio L., Bradley M.C., Dunn C.D. (2019). The endoplasmic Reticulum-Mitochondria encounter structure complex coordinates coenzyme Q biosynthesis. Contact (Thousand Oaks).

[12] Novales N.A., Meyer H., Asraf Y., Schuldiner M., Clarke C.F. (2025). Mitochondrial-ER contact sites and tethers influence the biosynthesis and function of coenzyme Q. Contact (Thousand Oaks).

[13] Subramanian K., Jochem A., Le Vasseur M., Lewis S., Paulson B.R., Reddy T.R. (2019). Coenzyme Q biosynthetic proteins assemble in a substrate-dependent manner into domains at ER-mitochondria contacts. J. Cell Biol..

[14] Bentinger M., Brismar K., Dallner G. (2007). The antioxidant role of coenzyme Q. Mitochondrion.

[15] Kagan V.E., Fabisiak J.P., Quinn P.J. (2000). Coenzyme Q and vitamin E need each other as antioxidants. Protoplasma.

[16] Hyun D.H., Hernandez J.O., Mattson M.P., de Cabo R. (2006). The plasma membrane redox system in aging. Ageing Res. Rev..

[17] Baschiera E., Sorrentino U., Calderan C., Desbats M.A., Salviati L. (2021). The multiple roles of coenzyme Q in cellular homeostasis and their relevance for the pathogenesis of coenzyme Q deficiency. Free Radic. Biol. Med..

[18] Fujii J., Yamada K.I. (2023). Defense systems to avoid ferroptosis caused by lipid peroxidation-mediated membrane damage. Free Radic. Res..

[19] Bersuker K., Hendricks J.M., Li Z., Magtanong L., Ford B., Tang P.H. (2019). The CoQ oxidoreductase FSP1 acts parallel to GPX4 to inhibit ferroptosis. Nature.

[20] Doll S., Freitas F.P., Shah R., Aldrovandi M., da Silva M.C., Ingold I. (2019). FSP1 is a glutathione-independent ferroptosis suppressor. Nature.

[21] Salviati L., Trevisson E., Doimo M., Navas P., Adam M.P., Mirzaa G.M., Pagon R.A., Wallace S.E., Bean L.J.H., Gripp K.W. (2017). Primary Coenzyme Q(10) Deficiency in GeneReviews((R)).

[22] Mantle D., Millichap L., Castro-Marrero J., Hargreaves I.P. (2023). Primary coenzyme Q10 deficiency: an update. Antioxidants (Basel).

[23] Mantle D., Turton N., Hargreaves I.P. (2022). Depletion and supplementation ofcoenzyme Q10 in secondary deficiency disorders. Front. Biosci. (Landmark Ed).

[24] Navas P., Cascajo M.V., Alcazar-Fabra M., Hernandez-Camacho J.D., Sanchez-Cuesta A., Rodriguez A.B.C. (2021). Secondary CoQ(10) deficiency, bioenergetics unbalance in disease and aging. BioFactors.

[25] Arenas-Jal M., Sune-Negre J.M., Garcia-Montoya E. (2020). Coenzyme Q10 supplementation: efficacy, safety, and formulation challenges. Compr. Rev. Food Sci. Food Saf..

[26] Pravst I., Rodriguez Aguilera J.C., Cortes Rodriguez A.B., Jazbar J., Locatelli I., Hristov H. (2020). Comparative bioavailability of different coenzyme Q10 formulations in healthy elderly individuals. Nutrients.

[27] Wang Y., Hekimi S. (2022). The efficacy of coenzyme Q(10) treatment in alleviating the symptoms of primary coenzyme Q(10) deficiency: a systematic review. J. Cell. Mol. Med..

[28] Guile M.D., Jain A., Anderson K.A., Clarke C.F. (2023). New insights on the uptake and trafficking of coenzyme Q. Antioxidants (Basel).

[29] Fernandez-Del-Rio L., Kelly M.E., Contreras J., Bradley M.C., James A.M., Murphy M.P. (2020). Genes and lipids that impact uptake and assimilation of exogenous coenzyme Q in *Saccharomyces cerevisiae*. Free Radic. Biol. Med..

[30] James A.M., Cocheme H.M., Murai M., Miyoshi H., Murphy M.P. (2010). Complementation of coenzyme Q-deficient yeast by coenzyme Q analogues requires the isoprenoid side chain. FEBS J..

[31] Fernandez-Ayala D.J., Brea-Calvo G., Lopez-Lluch G., Navas P. (2005). Coenzyme Q distribution in HL-60 human cells depends on the endomembrane system. Biochim. Biophys. Acta.

[32] Padilla-López S., Jiménez-Hidalgo M., Martín-Montalvo A., Clarke C.F., Navas P., Santos-Ocaña C. (2009). Genetic evidence for the requirement of the endocytic pathway in the uptake of coenzyme Q6 in Saccharomyces cerevisiae. Biochim. Biophys. Acta.

[33] Williams M., Kim K. (2014). From membranes to organelles: emerging roles for dynamin-like proteins in diverse cellular processes. Eur. J. Cell Biol..

[34] Arlt H., Raman B., Filali-Mouncef Y., Hu Y., Leytens A., Hardenberg R. (2023). The dynamin Vps1 mediates Atg9 transport to the sites of autophagosome formation. J. Biol. Chem..

[35] Shen T., Jiang L., Wang X., Xu Q., Han L., Liu S. (2021). Function and molecular mechanism of N-terminal acetylation in autophagy. Cell Rep..

[36] Cao W., Li J., Yang K., Cao D. (2021). An overview of autophagy: mechanism, regulation and research progress. Bull Cancer.

[37] Gomez-Virgilio L., Silva-Lucero M.D., Flores-Morelos D.S., Gallardo-Nieto J., Lopez-Toledo G., Abarca-Fernandez A.M. (2022). Autophagy: a key regulator of homeostasis and disease: an overview of molecular mechanisms and modulators. Cells.

[38] Feng Y., He D., Yao Z., Klionsky D.J. (2014). The machinery of macroautophagy. Cell Res..

[39] Zhen Y., Stenmark H. (2023). Autophagosome biogenesis. Cells.

[40] Li L., Tong M., Fu Y., Chen F., Zhang S., Chen H. (2021). Lipids and membrane-associated proteins in autophagy. Protein Cell.

[41] Kumar A.V., Mills J. (2023). Non-canonical autophagy in aging and age-related diseases. Front. Cell Dev. Biol..

[42] Munz C. (2021). Non-canonical roles of autophagy proteins in endocytosis and exocytosis. Biochem. Soc. Trans..

[43] Nieto-Torres J.L., Leidal A.M., Debnath J., Hansen M. (2021). Beyond autophagy: the expanding roles of ATG8 proteins. Trends Biochem. Sci..

[44] Reid S.E., Kolapalli S.P., Nielsen T.M., Frankel L.B. (2022). Canonical and non-canonical roles for ATG8 proteins in autophagy and beyond. Front. Mol. Biosci..

[45] Smaczynska-de Rooij I.I., Allwood E.G., Aghamohammadzadeh S., Hettema E.H., Goldberg M.W., Ayscough K.R. (2010). A role for the dynamin-like protein Vps1 during endocytosis in yeast. J. Cell Sci..

[46] Palmer S.E., Smaczynska-de Rooij I.I., Marklew C.J., Allwood E.G., Mishra R., Goldberg M.W. (2015). A charge swap mutation E461K in the yeast dynamin Vps1 reduces endocytic invagination. Commun. Integr. Biol..

[47] Palmer S.E., Smaczynska-de Rooij I.I., Marklew C.J., Allwood E.G., Mishra R., Johnson S. (2015). A dynamin-actin interaction is required for vesicle scission during endocytosis in yeast. Curr. Biol..

[48] Smaczynska-de Rooij I.I., Allwood E.G., Mishra R., Booth W.I., Aghamohammadzadeh S., Goldberg M.W. (2012). Yeast dynamin Vps1 and amphiphysin Rvs167 function together during endocytosis. Traffic.

[49] Smaczynska-de Rooij I.I., Marklew C.J., Allwood E.G., Palmer S.E., Booth W.I., Mishra R. (2015). Phosphorylation regulates the endocytic function of the yeast dynamin-related protein Vps1. Mol. Cell Biol..

[50] Smaczynska-de Rooij I.I., Marklew C.J., Palmer S.E., Allwood E.G., Ayscough K.R. (2019). Mutation of key lysine residues in the insert B region of the yeast dynamin Vps1 disrupts lipid binding and causes defects in endocytosis. PLoS One.

[51] Song B.D., Leonard M., Schmid S.L. (2004). Dynamin GTPase domain mutants that differentially affect GTP binding, GTP hydrolysis, and clathrin-mediated endocytosis. J. Biol. Chem..

[52] Song B.D., Yarar D., Schmid S.L. (2004). An assembly-incompetent mutant establishes a requirement for dynamin self-assembly in clathrin-mediated endocytosis in vivo. Mol. Biol. Cell.

[53] Caesar R., Warringer J., Blomberg A. (2006). Physiological importance and identification of novel targets for the N-terminal acetyltransferase NatB. Eukaryot. Cell.

[54] Guimaraes R.S., Delorme-Axford E., Klionsky D.J., Reggiori F. (2015). Assays for the biochemical and ultrastructural measurement of selective and nonselective types of autophagy in the yeast Saccharomyces cerevisiae. Methods.

[55] Torggler R., Papinski D., Kraft C. (2017). Assays to monitor autophagy in Saccharomyces cerevisiae. Cells.

[56] Shih C.H., Fay J. (2021). Cis-regulatory variants affect gene expression dynamics in yeast. eLife.

[57] Cebollero E., Reggiori F. (2009). Regulation of autophagy in yeast Saccharomyces cerevisiae. Biochim. Biophys. Acta.

[58] McCullough J., Frost A., Sundquist W.I. (2018). Structures, functions, and dynamics of ESCRT-III/Vps4 membrane remodeling and fission complexes. Annu. Rev. Cell Dev. Biol..

[59] Kihara A., Noda T., Ishihara N., Ohsumi Y. (2001). Two distinct Vps34 phosphatidylinositol 3-kinase complexes function in autophagy and carboxypeptidase Y sorting in Saccharomyces cerevisiae. J. Cell Biol..

[60] Lee H.N., Zarza X., Kim J.H., Yoon M.J., Kim S.H., Lee J.H. (2018). Vacuolar trafficking protein VPS38 is dispensable for autophagy. Plant Physiol..

[61] Aksnes H., Hole K., Arnesen T. (2015). Molecular, cellular, and physiological significance of N-terminal acetylation. Int. Rev. Cell Mol. Biol..

[62] Zientara-Rytter K., Subramani S. (2020). Mechanistic insights into the role of Atg11 in selective autophagy. J. Mol. Biol..

[63] Wilsbach K., Payne G.S. (1993). Vps1p, a member of the dynamin GTPase family, is necessary for Golgi membrane protein retention in Saccharomyces cerevisiae. EMBO J..

[64] Nothwehr S.F., Conibear E., Stevens T.H. (1995). Golgi and vacuolar membrane proteins reach the vacuole in vps1 mutant yeast cells via the plasma membrane. J. Cell Biol..

[65] Peters C., Baars T.L., Buhler S., Mayer A. (2004). Mutual control of membrane fission and fusion proteins. Cell.

[66] Holzer E., Martens S., Tulli S. (2024). The role of ATG9 vesicles in autophagosome biogenesis. J. Mol. Biol..

[67] Aksnes H., Drazic A., Marie M., Arnesen T. (2016). First things first: vital protein marks by N-Terminal acetyltransferases. Trends Biochem. Sci..

[68] Aksnes H., Ree R., Arnesen T. (2019). Co-translational, post-translational, and non-catalytic roles of N-Terminal acetyltransferases. Mol. Cell.

[69] Malhotra V. (2013). Unconventional protein secretion: an evolving mechanism. EMBO J..

[70] Duran J.M., Anjard C., Stefan C., Loomis W.F., Malhotra V. (2010). Unconventional secretion of Acb1 is mediated by autophagosomes. J. Cell Biol..

[71] Bruns C., McCaffery J.M., Curwin A.J., Duran J.M., Malhotra V. (2011). Biogenesis of a novel compartment for autophagosome-mediated unconventional protein secretion. J. Cell Biol..

[72] Millard E., Tooze S.A. (2025). ATG9 not just an autophagy related protein. J. Mol. Biol..

[73] New J., Thomas S.M. (2019). Autophagy-dependent secretion: mechanism, factors secreted, and disease implications. Autophagy.

[74] Ouahoud S., Fiet M.D., Martinez-Montanes F., Ejsing C.S., Kuss O., Roden M. (2018). Lipid droplet consumption is functionally coupled to vacuole homeostasis independent of lipophagy. J. Cell Sci..

[75] Zhang A., Meng Y., Li Q., Liang Y. (2020). The endosomal sorting complex required for transport complex negatively regulates Erg6 degradation under specific glucose restriction conditions. Traffic.

[76] Toulmay A., Prinz W.A. (2013). Direct imaging reveals stable, micrometer-scale lipid domains that segregate proteins in live cells. J. Cell Biol..

[77] Wang C.W., Miao Y.H., Chang Y.S. (2014). A sterol-enriched vacuolar microdomain mediates stationary phase lipophagy in budding yeast. J. Cell Biol..

[78] Tsuji T., Fujimoto M., Tatematsu T., Cheng J., Orii M., Takatori S. (2017). Niemann-pick type C proteins promote microautophagy by expanding raft-like membrane domains in the yeast vacuole. eLife.

[79] Tsuji T., Fujimoto T. (2018). Lipids and lipid domains of the yeast vacuole. Biochem. Soc. Trans..

[80] Rahman M.A., Kumar R., Sanchez E., Nazarko T.Y. (2021). Lipid droplets and their autophagic turnover via the raft-like vacuolar microdomains. Int. J. Mol. Sci..

[81] He C.W., Cui X.F., Ma S.J., Xu Q., Ran Y.P., Chen W.Z. (2021). Membrane recruitment of Atg8 by Hfl1 facilitates turnover of vacuolar membrane proteins in yeast cells approaching stationary phase. BMC Biol..

[82] Liu X.M., Yamasaki A., Du X.M., Coffman V.C., Ohsumi Y., Nakatogawa H. (2018). Lipidation-independent vacuolar functions of Atg8 rely on its noncanonical interaction with a vacuole membrane protein. eLife.

[83] Liparulo I., Bazhin A., Van Wyhe G.K., Gunawan A.L., Dadina N., Kwon J.H. (2026). A clickable CoQ imaging probe reveals that cellular uptake and lysosomal trafficking depend on CD36 and NPC1. Redox Biol..

[84] Wang Y., Hekimi S. (2025). Elevated cellular accumulation of endogenous and exogenous CoQ by altered intracellular trafficking. J. Biol. Chem..

[85] Nashimoto S., Takekawa Y., Takekuma Y., Sugawara M., Sato Y. (2020). Transport via Niemann-pick C1 like 1 contributes to the intestinal absorption of ubiquinone. Drug Metab. Pharmacokinet..

[86] Anderson C.M., Kazantzis M., Wang J., Venkatraman S., Goncalves R.L., Quinlan C.L. (2015). Dependence of brown adipose tissue function on CD36-mediated coenzyme Q uptake. Cell Rep..

[87] Deshwal S., Onishi M., Tatsuta T., Bartsch T., Cors E., Ried K. (2023). Mitochondria regulate intracellular coenzyme Q transport and ferroptotic resistance via STARD7. Nat. Cell Biol..

[88] Ndoci M., Bhattacharya S., Agrawal I., Hinze Y., Lemke K., Schumacher A.L. (2026). Mitochondria limit coenzyme Q export under cholesterol biosynthetic stress. J. Cell Biol..

[89] Pesini A., Barriocanal-Casado E., Compagnoni G.M., Hidalgo-Gutierrez A., Yanez G., Bakkali M. (2025). Coenzyme Q(10) deficiency disrupts lipid metabolism by altering cholesterol homeostasis in neurons. Free Radic. Biol. Med..

[90] Mizutani M., Kuroda S., Oku M., Aoki W., Masuya T., Miyoshi H. (2024). Identification of proteins involved in intracellular ubiquinone trafficking in Saccharomyces cerevisiae using artificial ubiquinone probe. Biochim. Biophys. Acta Bioenerg..

[91] Elbaz-Alon Y., Rosenfeld-Gur E., Shinder V., Futerman A.H., Geiger T., Schuldiner M. (2014). A dynamic interface between vacuoles and mitochondria in yeast. Dev. Cell.

[92] Crane F.L., Barr R., McCormick D.B., Wright L.D. (1971). Determination of Ubiquinones in *Vitamins and Coenzymes*.

[93] Brachmann C.B., Davies A., Cost G.J., Caputo E., Li J., Hieter P. (1998). Designer deletion strains derived from *Saccharomyces cerevisiae* S288C: a useful set of strains and plasmids for PCR-mediated gene disruption and other applications. Yeast.

[94] Burke D., Dawson D., Stearns T. (2000).

[95] McEwen J.E., Ko C., Kloeckner-Gruissem B., Poyton R.O. (1986). Nuclear functions required for cytochrome c oxidase biogenesis in Saccharomyces cerevisiae. Characterization of mutants in 34 complementation groups. J. Biol. Chem..

[96] Baudin A., Ozier-Kalogeropoulos O., Denouel A., Lacroute F., Cullin C. (1993). A simple and efficient method for direct gene deletion in Saccharomyces cerevisiae. Nucleic Acids Res..

[97] Gietz R.D., Woods R.A. (2002). Transformation of yeast by lithium acetate/single-stranded carrier DNA/polyethylene glycol method. Methods Enzymol..

[98] Tsui H.S., Pham N.V.B., Amer B.R., Bradley M.C., Gosschalk J.E., Gallagher-Jones M. (2019). Human COQ10A and COQ10B are distinct lipid-binding START domain proteins required for coenzyme Q function. J. Lipid Res..

[99] Zhang T., Lei J., Yang H., Xu K., Wang R., Zhang Z. (2011). An improved method for whole protein extraction from yeast Saccharomyces cerevisiae. Yeast.

[100] Schneider C.A., Rasband W.S., Eliceiri K.W. (2012). NIH Image to ImageJ: 25 years of image analysis. Nat. Methods.

[101] Banerjee S., Daetwyler S., Bai X., Michaud M., Jouhet J., Binns D. (2025). The Vps13-like protein BLTP2 regulates phosphatidylethanolamine levels to maintain plasma membrane fluidity and breast cancer aggressiveness. Nat. Cell Biol..

[102] Li Y., Prinz W.A. (2004). ATP-binding cassette (ABC) transporters mediate nonvesicular, raft-modulated sterol movement from the plasma membrane to the endoplasmic reticulum. J. Biol. Chem..

[103] Winzeler E.A., Shoemaker D.D., Astromoff A., Liang H., Anderson K., Andre B. (1999). Functional characterization of the S. Cerevisiae genome by gene deletion parallel analysis. Science.

[104] Santos-Ocana C., Do T.Q., Padilla S., Navas P., Clarke C.F. (2002). Uptake of exogenous coenzyme Q and transport to mitochondria is required for *bc*1 complex stability in yeast *coq* mutants. J. Biol. Chem..

[105] Guan J., Stromhaug P.E., George M.D., Habibzadegah-Tari P., Bevan A., Dunn W.A. (2001). Cvt18/Gsa12 is required for cytoplasm-to-vacuole transport, pexophagy, and autophagy in Saccharomyces cerevisiae and Pichia pastoris. Mol. Biol. Cell.

[106] Sikorski R.S., Hieter P. (1989). A system of shuttle vectors and yeast host strains designed for efficient manipulation of DNA in Saccharomyces cerevisiae. Genetics.

[107] Reidick C., Boutouja F., Platta H.W. (2017). The class III phosphatidylinositol 3-kinase Vps34 in Saccharomyces cerevisiae. Biol. Chem..

[108] Juarez-Montiel M., Clark-Flores D., Tesillo-Moreno P., de la Vega-Camarillo E., Andrade-Pavon D., Hernandez-Garcia J.A. (2022). Vacuolar proteases and autophagy in phytopathogenic fungi: a review. Front. Fungal Biol..

