# Supplementary material for: Vps1 and select autophagic machinery are required for coenzyme Q uptake and trafficking to mitochondria
Source: J Biol Chem. 2026 Sep 23;302(11):113592. doi: 10.1016/j.jbc.2026.113592 (PMC13691570; doi:10.1016/j.jbc.2026.113592)
Supplement: Supplementary file 1 [file mmc1.docx]

**Title:** Vps1 and select autophagic machinery are required for coenzyme Q uptake and trafficking to mitochondria

**Authors:** Michael D Guile^1^, Kyle A Anderson^1^, Akash Jain^1^, Alexandre Toulmay^2^, William A. Prinz^2^, and Catherine F Clarke^1,*^

^1^ Department of Chemistry and Biochemistry, University of California, Los Angeles, CA

^2^Department of Cell Biology, University of Texas Southwestern Medical Center, Dallas, TX

^*^ Corresponding author, email: cfclarke@ucla.edu

**Contents of Supporting Information:**

**Table S1**

**Table S2**

**Table S3**

**Table S4**

**Figure S1**

**Figure S2**

**Figure S3**

**Figure S4**

**Figure S5**

| **Table S1: Genotype and source of yeast strains** | | | |
| --- | --- | --- | --- |
| **Yeast Strain** | **Genetic Background** | **Genotype** | **Reference** |
| WT | BY4741 | *MAT a his3Δ1 leu2Δ0 met15Δ0 ura3Δ0* | (93) |
| *atg1Δ* | BY4741 | *MAT a his3Δ1 leu2Δ0 met15Δ0 ura3Δ1 atg1::KanMX* | (103) |
| *atg2Δ* | BY4741 | *MAT a his3Δ1 leu2Δ0 met15Δ0 ura3Δ1 atg2::KanMX* | (103) |
| *atg4Δ* | BY4741 | *MAT a his3Δ1 leu2Δ0 met15Δ0 ura3Δ1 atg4::KanMX* | (103) |
| *atg6Δ* | BY4741 | *MAT a his3Δ1 leu2Δ0 met15Δ0 ura3Δ1 atg6::KanMX* | (103) |
| *atg7Δ* | BY4741 | *MAT a his3Δ1 leu2Δ0 met15Δ0 ura3Δ1 atg7::KanMX* | (103) |
| *atg9Δ* | BY4741 | *MAT a his3Δ1 leu2Δ0 met15Δ0 ura3Δ1 atg9::KanMX* | (103) |
| *atg11Δ* | BY4741 | *MAT a his3Δ1 leu2Δ0 met15Δ0 ura3Δ1 atg11::KanMX* | (103) |
| *atg12Δ* | BY4741 | *MAT a his3Δ1 leu2Δ0 met15Δ0 ura3Δ1 atg12::KanMX* | (103) |
| *atg16Δ* | BY4741 | *MAT a his3Δ1 leu2Δ0 met15Δ0 ura3Δ1 atg16::KanMX* | (103) |
| *atg32Δ* | BY4741 | *MAT a his3Δ1 leu2Δ0 met15Δ0 ura3Δ1 atg32::KanMX* | (103) |
| *atg36Δ* | BY4741 | *MAT a his3Δ1 leu2Δ0 met15Δ0 ura3Δ1 atg36::KanMX* | (103) |
| *coq2Δ* | BY4741 | *MAT a his3Δ1 leu2Δ0 met15Δ0 ura3Δ1 coq2::NatMX* | This study |
| *coq2Δ* | BY4741 | *MAT a his3Δ1 leu2Δ0 met15Δ0 ura3Δ1 coq2::LEU2* | This study |
| *coq2Δ* | BY4741 | *MAT a his3Δ1 leu2Δ0 met15Δ0 ura3Δ1 coq2::KanMX* | (103) |
| JM6 | BY4741 | *MAT a his-4 ρ^0^* | (104) |
| JM8 | BY4742 | *MAT α ade-1 ρ^0^* | (104) |
| *nat3Δ* | BY4741 | *MAT a his3Δ1 leu2Δ0 met15Δ0 ura3Δ1 nat3::KanMX* | (103) |
| *pep4Δ* | BY4741 | *MAT a his3Δ1 leu2Δ0 met15Δ0 ura3Δ1 pep4::KanMX* | (103) |
| *vps1Δ* | BY4741 | *MAT a his3Δ1 leu2Δ0 met15Δ0 ura3Δ1 vps1::KanMX* | (103) |
| *vps4Δ* | BY4741 | *MAT a his3Δ1 leu2Δ0 met15Δ0 ura3Δ1 vps4::KanMX* | (103) |
| *vps38Δ* | BY4741 | *MAT a his3Δ1 leu2Δ0 met15Δ0 ura3Δ1 vps38::KanMX* | (103) |
| *atg1Δcoq2Δ* | BY4741 | *MAT a his3Δ1 leu2Δ0 met15Δ0 ura3Δ1 atg1::KanMX coq2::LEU2* | This study |
| *atg2Δcoq2Δ* | BY4741 | *MAT a his3Δ1 leu2Δ0 met15Δ0 ura3Δ1 atg2::KanMX coq2::LEU2* | This study |
| *atg4Δcoq2Δ* | BY4741 | *MAT a his3Δ1 leu2Δ0 met15Δ0 ura3Δ1 atg4::KanMX coq2::LEU2* | This study |
| *atg6Δcoq2Δ* | BY4741 | *MAT a his3Δ1 leu2Δ0 met15Δ0 ura3Δ1 atg6::KanMX coq2::NatMX* | This study |
| *atg7Δcoq2Δ* | BY4741 | *MAT a his3Δ1 leu2Δ0 met15Δ0 ura3Δ1 atg7::KanMX coq2::LEU2* | This study |
| *atg9Δcoq2Δ* | BY4741 | *MAT a his3Δ1 leu2Δ0 met15Δ0 ura3Δ1 atg9::KanMX coq2::LEU2* | This study |
| *atg11Δcoq2Δ* | BY4741 | *MAT a his3Δ1 leu2Δ0 met15Δ0 ura3Δ1 atg11::KanMX coq2::LEU2* | This study |
| *atg12Δcoq2Δ* | BY4741 | *MAT a his3Δ1 leu2Δ0 met15Δ0 ura3Δ1 atg12::KanMX coq2::LEU2* | This study |
| *atg16Δcoq2Δ* | BY4741 | *MAT a his3Δ1 leu2Δ0 met15Δ0 ura3Δ1 atg16::KanMX coq2::LEU2* | This study |
| *atg32Δcoq2Δ* | BY4741 | *MAT a his3Δ1 leu2Δ0 met15Δ0 ura3Δ1 atg32::KanMX coq2::LEU2* | This study |
| *atg36Δcoq2Δ* | BY4741 | *MAT a his3Δ1 leu2Δ0 met15Δ0 ura3Δ1 atg36::KanMX coq2::LEU2* | This study |
| *pep4Δcoq2Δ* | BY4741 | *MAT a his3Δ1 leu2Δ0 met15Δ0 ura3Δ1 pep4::KanMX coq2::LEU2* | This study |
| *nat3Δcoq2Δ* | BY4741 | *MAT a his3Δ1 leu2Δ0 lys2Δ ura3Δ can1::LEU2^+^ - MFA1pr-HIS3 nat3::KanMX coq2::natR* | (29) |
| *vps1Δcoq2Δ* | BY4741 | *MAT a his3Δ1 leu2Δ0 lys2Δ ura3Δ can1::LEU2^+^ - MFA1pr-HIS3 vps1::KanMX coq2::natR* | (29) |
| *vps4Δcoq2Δ* | BY4741 | *MAT a his3Δ1 leu2Δ0 met15Δ0 ura3Δ1 vps4::KanMX coq2::LEU2* | This study |
| *vps38Δcoq2Δ* | BY4741 | *MAT a his3Δ1 leu2Δ0 met15Δ0 ura3Δ1 vps38::KanMX coq2::LEU2* | This study |
| *atg1Δ vps1Δcoq2Δ* | BY4741 | *MAT a his3Δ1 leu2Δ0 lys2Δ ura3Δ can1::LEU2^+^ - MFA1pr-HIS3 atg1::URA3 vps1::KanMX coq2::natR* | This study |
| *atg9Δ vps1Δcoq2Δ* | BY4741 | *MAT a his3Δ1 leu2Δ0 lys2Δ ura3Δ can1::LEU2^+^ - MFA1pr-HIS3 atg9::URA3 vps1::KanMX coq2::natR* | This study |

| **Table S2: Plasmids used in this study** | | | |
| --- | --- | --- | --- |
| **Plasmid** | **Alias** | **Description** | **Reference** |
| pGFP-Atg8 | GFP-ATG8 | pRS416 with *GFP-ATG8* | (105) |
| pKA544 | EV | URA3, CEN with PGKterm | (47) |
| pKA677 | WT-Vps1 | pKA544 with *VPS1* under its own promoter for *in vivo* expression (inc 320 bp 5') | (34, 47) |
| pKA677-D2M | D2M | pKA544 with *vps1* D2M | This study |
| pKA677-D2P | D2P | pKA544 with *vps1* D2P | This study |
| pKA699 | T63A | pKA544 with *vps1* T63A | (34, 45) |
| pKA701 | I649K | pKA544 with *vps1* I649K | (34, 45) |
| pKA781 | S599V | pKA544 with *vps1* S599V | (34, 49) |
| pKA945 | E461K | pKA544 with *vps1* E461K | (34, 47, 46) |
| pKA760 | P564A | pKA544 with *vps1* P564A | (48) |
| pKA1203 | KKKAAA | pKA544 with *vps1* KKK591-593AAA | (50) |
| pRS305 | - | *LEU2* | (106) |
| pRS316 | EV | *URA3*, CEN | (29) |
| pRSQ2 | COQ2 | pRS316 with *COQ2* | (29) |

| **Table S3: Primers used in this study** | |
| --- | --- |
| Primer | Primer Sequence (5’ 🡪 3’) |
| *COQ2* Upstream Forward | GTTCTCCATTTAACCACAAAGTGTC |
| *COQ2* Downstream Reverse | AAACACGTAACGTCAAGACTAGTCA |
| *LEU2* Internal Forward | CCAGAAGTGCAAAGAATCACAA |
| *LEU2* Internal Reverse | TTGTGATTCTTTGCACTTCTGG |
| *LEU2* Forward with *COQ2* Overhang | CGGCATCGTGGAAAAGTTTCACAAGAATCAGGCCAAAAT  TGGCGCCTGATTCAAGAAATA |
| *LEU2* Forward with *COQ2* Overhang | AAGCCCAAATAGGTACGGACATCTACGTAAGAATGCATG  TATGCCCTCCTCCTTGTCAATA |
| *natMX6* Internal Forward | GCAGAAAGTAATATCATGCGTCA |
| *natMX6* Internal Reverse | GGTACCCATGGTTGTTTATGTTC |
| *URA3* Forward with *ATG1* Overhang | AAATCTCTTTTACAACACCAGACGAGAAATTAAGAAA  GCTTGTCTGTAAGCGGATGCC |
| *URA3* Reverse with *ATG1* Overhang | CATTTGTACTTAATAAGAAAACCATATTATGCATCAC  ACGTTTACAATTTCCTGATGCGGT |
| *URA3* Forward with *ATG9* Overhang | ACAGCCTGAAATATCAAAATCACGGAATTATTAGGTT  GCTTGTCTGTAAGCGGATGCC |
| *URA3* Reverse with *ATG9* | ATAGTTATATTGGATGATGTACACGACACAGTCTGCC  ACGTTTACAATTTCCTGATGCGGT |
| *VPS1 D2M* Forward | AAAACTGCACATTTTATATTATCAGATATCATGATGGAGCA  TTTAATTTCTACTATTAACAAGCTTCAG |
| *VPS1 D2M* Reverse | CTGAAGCTTGTTAATAGTAGAAATTAAATGCTCCATCATGA  TATCTGATAATATAAAATGTGCAGTTTT |
| *VPS1 D2P* Forward | AAAACTGCACATTTTATATTATCAGATATCATGCCTGAGCAT  TTAATTTCTACTATTAACAAGCTTCAG |
| *VPS1 D2P* Reverse | CTGAAGCTTGTTAATAGTAGAAATTAAATGCTCAGGCATGA  TATCTGATAATATAAAATGTGCAGTTTT |

| **Table S4: Antibodies used in this study** | | | | |
| --- | --- | --- | --- | --- |
| **Target protein** | **Antibody Name** | **Host Species** | **Dilution** | **Source** |
| Dpm1 | Anti-Dpm1 | Mice | 1:1,000 | Invitrogen |
| GFP | anti-Green Fluorescent Protein (GFP) | Chicken | 1:5,000 | Aves Labs |
| Mdh1 | anti-Mdh1 | Rabbit | 1:10,000 | ^a^ L. McAlister-Henn |
| Pma1 | anti-Pma1 | Rabbit | 1:5,000 | R. Serrano |
| Por1 | anti-Por1 | Mice | 1:1,000 | Invitrogen |
| Vph1 | Anti-Vph1 | Mice | 1:1,000 | Invitrogen |
| Vps1 | anti-Vps1 | Rat | 1:10,000 | ^b^ K. Ayscough |
| Chicken | IRDye® 800CW Donkey anti-Chicken | Donkey | 1:20,000 | LICORbio |
| Mice | IRDye® 680RD Donkey anti-Mice IgG | Donkey | 1:10,000 | LICORbio |
| Rat | IRDye® 680RD Goat anti-Rat IgG | Goat | 1:20,000 | LICORbio |
| Rabbit | IRDye® 680RD Goat anti-Rabbit IgG | Goat | 1:20,000 | LICORbio |
| Rabbit | IRDye® 800CW Donkey anti-Rabbit IgG | Donkey | 1:10,000 | LICORbio |
| ^a^ Dr. L. McAlister-Henn, Department of Molecular Biophysics and Biochemistry, University of Texas Health Sciences Center, San Antonio, TX.  ^b^ Dr. K. Ayscough, Department of Biomedical Sciences, University of Sheffield, England | | | | |

**Figure S1: Vps1 is required for efficient CoQ_6_ trafficking in non-fermentable medium.** A) The OD_600_ of strains grown in fermentable medium (YPD) with or without 2 µM CoQ_6_ was measured over seven days with an initial OD_600_ of ~0.2. B) The terminal OD_600_ after seven days of growth from panel A is shown. Statistics were determined via two-way ANOVA with Šidák’s multiple comparisons tests. C) After seven days of growth as described in panel A, cells were serially diluted and spot-plated onto YPD and then incubated for two days to verify viability. One of three biological replicates is shown. D) The cell-associated CoQ_6_ content was determined from lipid extracts of whole cells after seven days of growth as described in panel A via HPLC-MS. Statistics were determined via an unpaired t-test. E) The cell-associated CoQ_6_ content was determined at the indicated times after growth as described in panel A. Statistics were determined via two-way ANOVA with Šidák’s multiple comparisons tests. *, p ≤ 0.05; **, p ≤ 0.01; ***, p ≤ 0.001; and ****, p ≤ 0.0001; ns, p > 0.05; ND indicates CoQ_6_ content was below detection limit.

**Figure S2: Growth curves with *vps1* point mutant plasmids.** The Abs_600nm_ of strains grown in YPGG with or without 2 µM CoQ_6_ (Q_6_) was measured over seven days. Cultures were started with an initial Abs_600nm_ of ~0.6. The final Abs _600 nm_ after seven days of growth is reported in figures 3 and 4. N = 3.

 **Figure S3: Vps1 is required for efficient autophagy.** A) Cells containing the pGFP-Atg8 plasmid were grown to log phase in selective medium (SD –Ura) and then transferred to nitrogen-starvation medium (SD –N) for six hours. Proteins were precipitated, and samples were prepared for immunoblot analysis. Aliquots of whole-cell lysates containing 25 µg protein were subjected to 12% Tris-glycine SDS-PAGE, transferred to a PVDF membrane, and probed with antisera to GFP. A representative immunoblot (one of three biological replicates) is shown. B) Band intensities corresponding to GFP-Atg8 and GFP were quantified by hand using ImageJ and the GFP band was plotted as a ratio of cleaved GFP peak area/total GFP peak area. The data show the mean ± the standard deviation of three biological replicates. Statistics were determined via one-way ANOVA with Tukey’s multiple comparisons test. p ≤ 0.0001.

**Figure S4: Restoration of respiration of *orfΔcoq2Δ* mutants upon plasmid expression of *COQ2*.** Strains were transformed with either pRSQ2 containing the wild type yeast *COQ2* gene or with the empty vector pRS316 as a negative control. Successful transformants were cultured in SD -ura overnight and 5 serial dilutions were plated on YPD, SD -ura, and YPG plates. The ability of *COQ2* to restore respiration through restoration of CoQ_6_ biosynthesis is observed in the last row of each YPG plate. Plates were incubated at 30 °C for 3-4 days.

**Figure S5: Autophagy mutants are defective for autophagy. A)** Cells containing the pGFP-Atg8 plasmid were grown to log phase in selective medium (SD -ura) and then transferred to nitrogen-starvation medium (SD -N) for six hours. Proteins were precipitated, and samples were prepared for immunoblot analysis. Aliquots of whole-cell lysates containing 25 µg protein were subjected to 12% Tris-glycine SDS-PAGE, transferred to a PVDF membrane, and probed with antisera to GFP. A representative immunoblot (one of three biological replicates) is shown. B) Band intensities corresponding to GFP-Atg8 and GFP were quantified by hand using ImageJ and the GFP band was plotted as a ratio of GFP peak area/total peak area. The data show the mean ± the standard deviation of three biological replicates. Genes that are essential for CoQ­_6_ uptake and trafficking are bolded. Asterisks denote the different degrees of significance as determined by unpaired t-tests between the *orfΔcoq2Δ* mutants and *coq2Δ*. *, p ≤ 0.05; **, p ≤ 0.01; ***, p ≤ 0.001; and ****, p ≤ 0.0001; NS, p > 0.05.
